# The potential of remdesivir to affect function, metabolism and proliferation of cardiac and kidney cells in vitro

**DOI:** 10.1007/s00204-022-03306-1

**Published:** 2022-05-17

**Authors:** Katja Merches, Leonie Breunig, Julia Fender, Theresa Brand, Vanessa Bätz, Svenja Idel, Laxmikanth Kollipara, Yvonne Reinders, Albert Sickmann, Angela Mally, Kristina Lorenz

**Affiliations:** 1 Institute of Pharmacology and Toxicology, University of Würzburg, Würzburg, Germany; 2 Present Address: Landesamt für Gesundheit und Lebensmittelsicherheit (LGL), Erlangen, Germany; 3 Leibniz-Institut für Analytische Wissenschaften-ISAS-e.V., Dortmund, Germany; 4 Department of Chemistry, College of Physical Sciences, University of Aberdeen, Aberdeen, Scotland, UK; 5 Medizinische Fakultät, Medizinisches Proteom-Center (MPC), Ruhr-Universität Bochum, Bochum, Germany; 6 PGS Toxicology and Environmental Protection, University of Leipzig, Johannisallee 28, Leipzig, Germany

**Keywords:** Remdesivir, Cardiotoxicity, Nephrotoxicity, Mitochondrial toxicity, COVID-19, Nucleoside analog

## Abstract

Remdesivir is a prodrug of a nucleoside analog and the first antiviral therapeutic approved for coronavirus disease. Recent cardiac safety concerns and reports on remdesivir-related acute kidney injury call for a better characterization of remdesivir toxicity and understanding of the underlying mechanisms. Here, we performed an in vitro toxicity assessment of remdesivir around clinically relevant concentrations (*C*_max_ 9 µM) using H9c2 rat cardiomyoblasts, neonatal mouse cardiomyocytes (NMCM), rat NRK-52E and human RPTEC/TERT1 cells as cell models for the assessment of cardiotoxicity or nephrotoxicity, respectively. Due to the known potential of nucleoside analogs for the induction of mitochondrial toxicity, we assessed mitochondrial function in response to remdesivir treatment, early proteomic changes in NMCM and RPTEC/TERT1 cells and the contractile function of NMCM. Short-term treatments (24 h) of H9c2 and NRK-52E cells with remdesivir adversely affected cell viability by inhibition of proliferation as determined by significantly decreased ^3^H-thymidine uptake. Mitochondrial toxicity of remdesivir (1.6–3.1 µM) in cardiac cells was evident by a significant decrease in oxygen consumption, a collapse of mitochondrial membrane potential and an increase in lactate secretion after a 24–48-h treatment. This was supported by early proteomic changes of respiratory chain proteins and intermediate filaments that are typically involved in mitochondrial reorganization. Functionally, an impedance-based analysis showed that remdesivir (6.25 µM) affected the beat rate and contractility of NMCM. In conclusion, we identified adverse effects of remdesivir in cardiac and kidney cells at clinically relevant concentrations, suggesting a careful evaluation of therapeutic use in patients at risk for cardiovascular or kidney disease.

**Supplementary Information:**

The online version contains supplementary material available at 10.1007/s00204-022-03306-1.

## Introduction

Remdesivir (GS-5734) is the first antiviral drug approved for COVID-19, a current global pandemic disease caused by severe acute respiratory syndrome-coronavirus-2 (SARS-CoV-2). Remdesivir (Veklury^®^) has a conditional marketing authorization in the European Union (EMA 2020) and an emergency use authorization in the USA since 2020 for patients above the age of 12 years requiring supplemental oxygen. Its antiviral activity was shown in vitro for different RNA viruses, like different SARS viruses, middle-east respiratory syndrome (MERS) virus, different zoonotic and human coronaviruses and Ebola virus (Eastman et al. 2020; Gordon et al. 2020a).

Remdesivir (GS-5734) is highly effective against SARS-CoV-2 replication in vitro and in animals (EMA 2020; Pruijssers et al. 2020; Wang et al. 2020a). It is a cell-permeable diastereomeric monophosphoramidate prodrug of a 1′-cyano-substituted adenine C-nucleoside ribose analog (Siegel et al. 2017). GS-5734 forms the intermediate alanine metabolite GS-704277, which is transformed to the nucleoside GS-441524 or its monophosphate. Both are further phosphorylated to the pharmacologically active nucleoside triphosphate GS-443902 by intracellular kinases (Hu et al. 2020; Xie and Wang 2021; Yan and Muller 2020). The triphosphate inhibits the RNA-dependent RNA polymerase (RdRp) of SARS-CoV-2 and leads to delayed chain termination in the replication process (Gordon et al. 2020b; Kokic et al. 2021).

The efficacy of remdesivir in COVID-19 patients remains controversial. Initial studies found associations with a faster recovery with remdesivir treatment (Beigel et al. 2020; Wang et al. 2020b). The SOLIDARITY trial initiated by the world health organization (WHO) showed that despite some benefits of remdesivir in low-risk patients the mortality rate was not significantly improved so that its use is not recommended by the WHO as COVID-19 treatment (Pan et al. 2021). However, an analysis of viral loads in hospitalized patients revealed a significantly faster viral clearance (by a median of 0.7 days) after remdesivir treatment (Lingas et al. 2022). Consistent with the assumption that inhibition of viral replication at the beginning of a disease is most effective (Lega et al. 2020), a retrospective observational study found a significant reduction of length of stay in hospital by 3.6 days, when patients were treated with remdesivir < 3 days after a positive test compared to patients treated at later disease stages (Paranjape et al. 2021). A placebo-controlled study with non-hospitalized COVID-19 patients at risk for severe disease revealed a 87% lower risk for hospitalization or death after remdesivir treatment (Gottlieb et al. 2022). However, due to concerns of drug-related toxicity remdesivir has been approved only for severely ill patients (EMA 2020). Recently, the US Food and Drug Administration (FDA) expanded the approval of remdesivir for the treatment of outpatients with mild to moderate disease and also the European Medicines Agency (EMA) is considering such action (EMA 2021a; FDA 2022).

Preclinical toxicity studies revealed considerable nephrotoxicity of remdesivir in rats and monkeys, which led to the restriction of remdesivir treatment to patients with intact kidney function (EMA 2020). However, a comprehensive analysis of remdesivir-related nephrotoxicity by the pharmacovigilance risk assessment committee (PRAC) of the EMA, which was conducted due to emerging clinical reports (Gérard et al. 2021), found no significant association of nephrotoxicity and remdesivir treatment based on the currently available data (EMA 2021b). Despite the absence of significantly increased total adverse events observed during treatment time in clinical studies (Beigel et al. 2020; Wang et al. 2020b), there is some evidence for adverse cardiac effects in patients given remdesivir. Among the 175 patients receiving remdesivir in the NCT03719586 Ebola trial, there was one severe case with hypotension and cardiac arrest (Mulangu et al. 2019). This prompted Aggarwal et al. in May 2020 to recommend thorough monitoring of cardiovascular safety in remdesivir-treated patients (Aggarwal et al. 2020). A systematic analysis of case-reports from 6574 COVID-19 patients among whom 2603 received remdesivir revealed 11.6% cardiac events in remdesivir-treated patients, significantly more than in patients treated with hydroxochloroquine, lopinavir/ritonavir, tocilizumab or glucocorticoids. Of these cardiac events, 31% were reports on bradycardia (Touafchia et al. 2021). Another analysis of a case-report-dataset revealed a two-fold increased risk for cardiac adverse events after remdesivir treatment (Rafaniello et al. 2021). Small cohort studies showed a significant association between remdesivir treatment and transient bradycardia (Adamo et al. 2022; Pallotto et al. 2021). Based on these results, the PRAC started a new safety signal procedure for cardiotoxicity (EMA 2021b).

The understanding of the underlying toxicity mechanisms of remdesivir is important for future treatment strategies and drug development. So far, only a few in vitro studies assessed the toxic mechanisms of remdesivir. These studies suggest that the formation of the nucleoside-triphosphate by adenylate kinase 2 is essential for cytotoxicity (Akinci et al. 2020; Monteil et al. 2020) and that its intracellular concentration, which is cell type dependent, correlates with mitochondrial toxicity (Fišar et al. 2021; Xu et al. 2020). Of note, cardiomyocytes and proximal tubule cells are characterized by high mitochondrial activity leading to high sensitivity to mitochondrial toxins (Tang et al. 2021; Varga et al. 2015).

To study the impact of remdesivir on cardiac and kidney cells, we used two different cardiomyocyte cell types, H9c2 cardiomyoblasts and neonatal mouse cardiomyocytes (NMCM), and two different kidney cell lines, renal proximal tubular epithelial cells immortalized telomerase reverse transcriptase 1 (RPTEC/TERT1) and normal rat kidney cells (NRK-52E). H9c2 and NRK-52E cells are immature proliferative cells with a limited potential to predict specific cardio- or nephrotoxicity (Lechner 2014; Lin and Will 2012). However, these cells were included as animal-free cellular models that are often used for the assessment of cardio- or nephrotoxicity, respectively (Liu et al. 2012, 2014, 2015; Lund et al. 2007; Lund and Wallace 2004; Lynx et al. 2008). Since H9c2 and NRK-52E cells are rather immature cell lines with less mitochondria than described for respective primary cardiomyocytes or renal proximal tubular cells and because of the absence of spontaneous beating behavior, also NMCM and RPTEC/TERT1 were used as more valuable models for mitotoxicity and changes in beating behavior. We think that the combination of different cell lines can shed light on different aspects of toxicity.

Here, we employed these different cell models to assess the potential cardio- and nephrotoxic mechanisms of remdesivir, representatives of two organs at risk for adverse effects. Interestingly, cytotoxicity was rather related to anti-proliferative effects than to cell death and mitotoxic effects of remdesivir were especially evident in cells with high mitochondrial activity, i.e., the cardiac cell types and RPTEC/TERT1.

## Materials and methods

### Preparation and culture of neonatal mouse cardiomyocytes (NMCM) and fibroblasts

NMCM were isolated from 1 to 3-day-old FVB/N mice (Lorenz et al. 2009; Schmid et al. 2015) purchased from Janvier Labs (Le Genest-Saint-Isle, France) or Charles River Laboratories (Wilmington, MA, USA) using the Neonatal Heart Dissociation Kit (Miltenyi Biotec) for the gentle MACS Octo Dissociator (Miltenyi Biotec) according to manufacturer’s instructions. Cardiomyocytes were enriched to 80–90% by early sedimentation of fibroblasts at 37 °C, 1% CO_2_ twice for 45 min each at a density of 10–20 hearts per untreated 10 cm dish (Sarstedt). The culture plates for NMCM were pre-coated with 100 µg/mL poly-lysine (MP Biomedicals) in PBS for at least 30 min or with 10 µg/mL fibronectin (Sigma) in PBS for at least 1.5 h before final cell seeding.

NMCM were cultured in Minimal Essential Medium Eagle (MEM; Sigma) containing 100 U/mL penicillin, 100 μg/mL streptomycin, 2 mM l-glutamine (Sigma), 350 mg/L NaHCO_3_ (Applichem), 30 mg/L 5-bromo-2′-deoxyuridine (BrdU; Sigma), 2 mg/L vitamin B12 (Sigma), and 5% (V/V) fetal calf serum at 37 °C and 1% CO_2_. If not stated otherwise, FCS concentration was reduced to 1% (V/V) after 24 h and to 0% FCS after another 24 h. Treatment of NMCM was performed 72 h after isolation in 0% FCS MEM, unless stated otherwise. Further information on properties of NMCM are given in supplementary methods.

### Cell culture of H9c2, NRK-52E and RPTEC/TERT1 cells

The H9c2 (2–1) cell line (Sigma; RRID: CVCL_0286) was cultured in DMEM high glucose, with 1 mM sodium-pyruvate and 3.75 mM glutamine, supplemented with 10% (V/V) FCS, 100 U/mL penicillin and 100 µg/mL streptomycin at 37 °C and 5% CO_2 _(Tomasovic et al. 2020).  For the preparation of 25 mM and 5 mM glucose and 10 mM galactose media, DMEM without glucose (Gibco) was supplemented with 5 mM Hepes (Sigma), 1 mM sodium-pyruvate (Applichem) and d-(+)-glucose (Applichem) or d-(+)-galactose (Sigma).

NRK-52E cells (ECACC, No. 87012902; RRID:CVCL_0468) were cultured in DMEM high glucose, supplemented with 2 mM l-glutamine and 10 µM non-essential-amino-acids (Gibco), 5% (V/V) FCS, 100 U/mL penicillin and 100 µg/mL streptomycin at 37 °C and 5% CO_2_. For preparation of glucose and galactose media, DMEM without glucose was supplemented with 5% (V/V) FCS, 100 U/mL penicillin, 100 µg/mL streptomycin and 25 mM d-(+)-glucose or 10 mM d-(+)-galactose. For conditioning NRK-52E cells towards mitochondrial respiration for measurements of oxygen consumption rates, cells were cultured in an 1:1 mix of glucose- and galactose-medium for 5 days.

RPTEC/TERT1 cells (Evercyte; Ord. No. CHT-003-0002; unlimited license) were cultured in a 1:1 mixture of DMEM no glucose (Gibco) and Ham’s F12 (Gibco), supplemented with 10 ng/mL epidermal growth factor (EGF; Sigma), 10 ng/mL Glutamax (Thermo Fisher), 5 µg/mL of each insulin/transferrin/sodium selenite (ITS; Sigma), 36 µg/mL hydrocortisone (Sigma), 100 U/mL penicillin and 100 µg/mL streptomycin and 5% (V/V) FCS. For testing different metabolic conditions, 10 ng/mL EGF, 10 ng/mL Glutamax, 5 µg/mL of ITS, 36 µg/mL hydrocortisone, 100 U/mL penicillin, 100 µg/mL streptomycin, and 5% (V/V) FCS were added to DMEM-F12 (biowest) in addition to 25 mM d-(+)-glucose or 10 mM d-(+)-galactose.

Further information on properties of respective cell lines are given in supplementary methods.

### Viability assessment

Cells were treated with increasing concentrations of remdesivir (Cayman Chemical or MedChemExpress; purity > 98%; 0.8–400 µM), zidovudine (MedChemExpress, purity 98%; 0.8–1200 µM) or zalcitabine (Selleckchem, purity 100%; 2.4–1200 µM) for 24–120 h. Zidovudine and zalcitabine are known for mitotoxic effects during HIV therapy (White 2001). Zidovudine, but not zalcitabine, was shown to change the metabolism of H9c2 cells 6 days after treatment at 50 µM (Lund et al. 2007). Due to our own observation of zalcitabine reducing mitochondrial DNA in RPTEC/TERT1 cells after long term treatment (Fig. S5e), we compared remdesivir toxicity in cardiac cells or kidney cell lines to the acute toxicity of high doses zidovudine or zalcitabine, respectively. For positive control, cells were treated with high concentrations of dmso (up to 20%) or antimycin A (Sigma, up to 50 µM). ATP levels were determined as indicator of cell vitality of cells cultured to 70–100% confluence in 96-well plates by the CellTiter-Glo^®^ assay (promega) according to manufacturer’s instructions. Luminescence counts acquired by the synergy neo2 multi-mode reader using the software Gen5™ 3.10 (BioTek) were compared between treatment groups. Supernatants were stored to assess lactate concentrations and LDH-activity.

### Determination of relative cell numbers

10–100% confluent cell-monolayers in 96-well plates were fixed with 4% paraformaldehyde (Sigma) in PBS, followed by permeabilization with 0.2% Triton^®^ X-100 (Applichem) in PBS and staining with 0.35 ng/mL 4′,6-diamidino-2-phenylindole (DAPI; Molecular Probes). 4–8 pictures per well were automatically taken with a Leica DMi8 microscope and the software Leica Application Suite X 3.7.423463 (Leica microsystems). Image analysis was performed with Image J Fiji RRID: SRC_002285 (Schindelin et al. 2012). The following macro was used to reveal the % area covered by nuclei in each image:

run("8-bit");

run ("Invert");

run("Auto Threshold", "method = Li");

run("Measure");

### ^*3*^*H-thymidine incorporation*

On the day before treatment H9c2 or NRK-52E cells (40.000 per well) were seeded in a 24-well plate in standard culture medium. For treatment, the medium was exchanged for 200 µL of standard culture medium containing 0.25 µCi/mL methyl-^3^H-thymidine (PerkinElmer) and respective concentrations of remdesivir, 8-(4-chlorophenylthio)adenosine 3′,5′-cyclic monophosphate (8-CPT-cAMP; Abcam) or solvent control (dimethylsulfoxide (dmso); Sigma). Cells were incubated for 6 h at 37 °C, 0% CO_2_, followed by 3 washing steps with phosphate buffered saline (PBS), overnight incubation with 5% tricarboxylic acid (TCA; Applichem) and cell lysis with 0.5 mM NaOH (Applichem). Lysates were mixed with 4 mL Rotiszint^®^ eco plus (Roth) in 6.5 mL scintillation-tubes (Roth) and counts per minute were recorded by a Tri-Carb^®^ 2910 TR Low Activity Liquid Scintillation Analyzer (PerkinElmer). Each condition was measured in triplicates.

### Cell cycle analysis

40,000 NRK-52E cells were seeded in 24-well plates 24 h prior treatment. Cells were treated with remdesivir for 24 h. Supernatants and trypsinized cells were fixed with 70% ethanol and subsequently stained with 0.7 ng/mL DAPI in PBS. DAPI-intensities were recorded by flow cytometry on the BD Aria III flow cytometer and analyzed using the software BD FACSDiva 8.0.1 (BD biosciences). Measurements were performed in duplicates. Doublet cells were identified by forward scatter width (FSC-W) to area (FSC-A) comparison and excluded from the analysis. The percentages of cells in G1-, S-, or G2/M-phase were extracted from histograms according to the intensity of the signal from DNA-staining (Luo et al. 2020).

### Impedance measurements for the assessment of cardiomyocyte beating

NMCM were seeded at a density of 70.000 cells per well in a NSP96-2 mm 96-well plate with gold electrodes at the bottom (Nanion Technologies) (Maimari et al. 2019). Plates were pre-coated with 10 µg/mL fibronectin in PBS 1.5 h before seeding. After 24 h, initial 5% (V/V) FCS containing medium was exchanged to 200 µl of 1% (V/V) FCS medium and the plate was transferred into the Cardio Excyte incubator with the measurement platform (Nanion Technologies). Cells were monitored with the Cardio Excyte Control software for another 24 h at 37 °C and 1% CO_2_ and medium was changed to serum-free medium for another 24 h. The next day medium was changed to 100 µl of fresh serum-free medium. For treatment, remdesivir and controls were diluted in the respective culture medium for the respective day in culture and pre-incubated at 37 °C for 15 min. Cell monitoring was paused for approximately 2 min to add the diluted substances. Impedance values were acquired in 20-s intervals every 30 min for minimum 24 h after treatment. Values were averaged from 4 to 6 wells per group and wells with arrhythmic beating cells before the treatment were excluded from the analysis. Data from specific time points after treatment were normalized against the solvent control for the group of technical replicates using the software Cardio Excyte Control 96. The parameters base impedance, beat rate, pulse width, and beat amplitude were calculated by the Cardio Excyte Control 96 software package (Nanion Technologies).

### Lactate assay

Lactate concentrations in supernatants of cultured cells were measured by a colorimetric assay, in which lactate is converted into pyruvate under the consumption of nicotinamide adenine dinucleotide (NAD) (Babson and Phillips 1965; Limonciel et al. 2011). Reactive solutions were prepared on the basis of Limonciel et al. (2011). Triethanolamine (Sigma), ethylene diamine tetraacetic acid (EDTA; Roth), magnesium chloride (Applichem), *N*-methylphenazinium methosulfate (PMS; Sigma), iodonitrotetrazolium chloride (INT; Sigma), ethanol, Triton^®^X-100 (AppliChem), ß-nicotinamide-adenine-dinucleotide hydrate (NAD; Sigma) and lactate dehydrogenase (LDH; Sigma) were combined for the color solution. 10 µL of supernatant was mixed with 90 µL of color solution. For quantification, a standard curve with sodium-l-lactate (Sigma) from 50 to 0.2 mM was generated and lactate concentrations in supernatants were interpolated using non-linear-curve-fitting by GraphPad Prism 9.1.2 (GraphPad Software).

### Metabolic profiling

The metabolic profile of H9c2, NRK-52E and RPTEC/TERT1 cells was determined with the Seahorse technology (Agilent Technologies), whereby the oxygen consumption rate (OCR, in pmol/min) was calculated from measurements via O_2_-detecting fluorophores over time. The Seahorse XF Cell Mito Stress Test Kit was assayed in a 96-well format and processed according to the manufacturer’s instructions using the Seahorse XFe96 Analyzer (Agilent Technologies). Per treatment group, 7–8 technical replicates were analyzed. H9c2 or NRK-52E cells (20,000 cells/well) were seeded 24 h before treatment in standard culture medium and incubated at 37 °C and 5% CO_2_. NRK-52E cells were cultured in 12.5 mM glucose and 5 mM galactose-containing medium 5 days before measurement to elevate mitochondrial activity. RPTEC/TERT1 cells were seeded at a density of 20,000 cells per well 4 days before treatment in standard culture medium. 24 h after treatment the medium was exchanged for Seahorse XF DMEM medium (Agilent technologies) supplemented with 4.5 g/L glucose (H9c2) or 1.8 g/L glucose (RPTEC/TERT1) or 1.8 g/L galactose (NRK-52E), 2 mM glutamine and 1 mM sodium pyruvate (Applichem) plus 10 µM non-essential amino acids for NRK-52E cells. After a 1-h incubation at 37 °C, 0% CO_2_, OCR was measured for 3 min every 3 min. Subsequently, inhibitors of components of the respiratory chain were added to the cells. The order of inhibitors used was oligomycin (Sigma; 3 μM), carbonyl cyanide-*p*-trifluoromethoxyphenylhydrazone (FCCP, Sigma; 3.5 µM for H9c2, 3 µM for RPTEC/TERT1 and NRK-52E) and antimycin A/rotenone (AA/Rot, Sigma; 1.5 μM). Four basal measurements and three measurements after addition of each inhibitor (each 3 min mixing, 3 min measurement) were conducted. To test the reversibility of effects of remdesivir, OCR was measured for 3 min every 20 min continuously for 12 h without the addition of inhibitors. Data were normalized and analyzed with the Wave Desktop and Controller 2.6 Software, version 2.6.1 (Agilent Technologies). Normalization to cell number was performed after the measurement by the determination of the area fraction of DAPI-stained nuclei in the wells using image J Fiji RRID: SRC_002285 (Schindelin et al. 2012) and the Wave Desktop and Controller 2.6 Software. The parameters base OCR, ATP-linked respiration, proton-leak, maximal respiration and spare respiratory capacity were calculated from the exported OCR-values according to the manual of the Mito Stress Test (Agilent technologies).

### ROS assay

ROS production was assessed by measurement of H_2_O_2_ in supernatants of H9c2 and NRK-52E cells. To this end 15,000 H9c2 cells or 10.000 NRK-52E cells per well were seeded into a 96-well plate in standard culture medium 24 h prior to treatment. Cells were treated with remdesivir, the positive control antimycin A (Sigma) or the solvent dmso in culture medium containing 15 µM diethylenetriaminepentaacetic acid (DTPA, Sigma), 5 U/mL superoxide dismutase (SOD, Sigma), 1 U/mL horseradish peroxidase (HRP, Sigma) and 10 µM Amplex^®^UltraRed (Life Technologies) for 24 h. Fluorescence in the supernatant was measured at 540 nm excitation and 590 nm emission at the synergy neo2 multi-mode reader using the software Gen5™ 3.10 (BioTek). Measurements were performed in triplicates.

### Mitochondrial membrane potential

H9c2 cells and neonatal mouse cardiomyocytes were seeded on glass coverslips (coated with poly-lysine). Cells were incubated with remdesivir (6 and 12 µM, respectively) for 24 h and stained with 2.5 nM TMRM (tetramethylrhodamine methyl ester; Invitrogen) and 100 nM Mito Tracker™ Green FM (Invitrogen) for 1 h. For data acquisition, a Leica TCS SP5 confocal microscope (Leica microsystems) was used. Excitation of TMRM was performed at 561 nm and emission was detected between 580 and 700 nm. Excitation of MitoTrackerGreen was performed at 488 nm and emission was detected between 500 and 530 nm.

For analysis of mitochondrial membrane potential, at least 60 H9c2 cells and 30 NMCM for each experiment were analyzed in a blinded manner using the quantification option of Leica Application Suite X, version 3.5.6.21594. The TMRM-signal was assessed relative to the MitoTracker-signal.

### Label-free protein quantitation

Briefly, NMCM and RPTEC/TERT1 cells were pretreated with either dmso (control) or remdesivir (9 µM) or antimycin A (0.2 µM) and lysed with 1% SDS buffer containing 50 mM Tris–Cl, 150 mM NaCl, pH 7.8 with complete mini protease inhibitor cocktail (Sigma). Sample cleaning and proteolysis (trypsin) were performed using the S-Trap mini protocol as previously described (Hentschel et al. 2021). Tryptic peptides were quality controlled (Burkhart et al. 2012) followed by LC–MS analysis using an Ultimate 3000 nano RSLC system coupled to a Q Exactive HF mass spectrometer (both Thermo Scientific). Precursor-based label-free protein quantification was performed with the Proteome Discoverer 2.3 (Thermo Scientific) software. In both data sets, for each protein, the ratios i.e., remdesivir/dmso and antimycin A/dmso were calculated using the normalized and scaled abundances. Furthermore, log2-fold-change and t-test p-values (including Benjamini–Hochberg adjusted) were determined by the Proteome Discoverer as described in the supplemental methods.

### Statistical analysis

For statistical analysis, we used GraphPad Prism 9.1.2 (GraphPad Software, San Diego, USA). Data are presented as mean ± standard deviation. Independent experiments (*n*) were performed on different days and/or with different cell-batches. Unless stated otherwise, data were normalized to the solvent control (dmso) for each independent experiment and a Kruskal–Wallis test, followed by a Dunn’s test for multiple comparisons was used to determine statistical significance of deviation of normalized values from control. In case of two factors, a 2-way ANOVA and Sidak’s multiple comparison test were performed. Degrees of significance from multiple comparison tests are indicated within the graphs by asterisks (*p < 0.05, **p < 0.01, ***p < 0.001).

## Results

### Cytotoxicity of remdesivir in cardiac cells

**Fig. 1:**
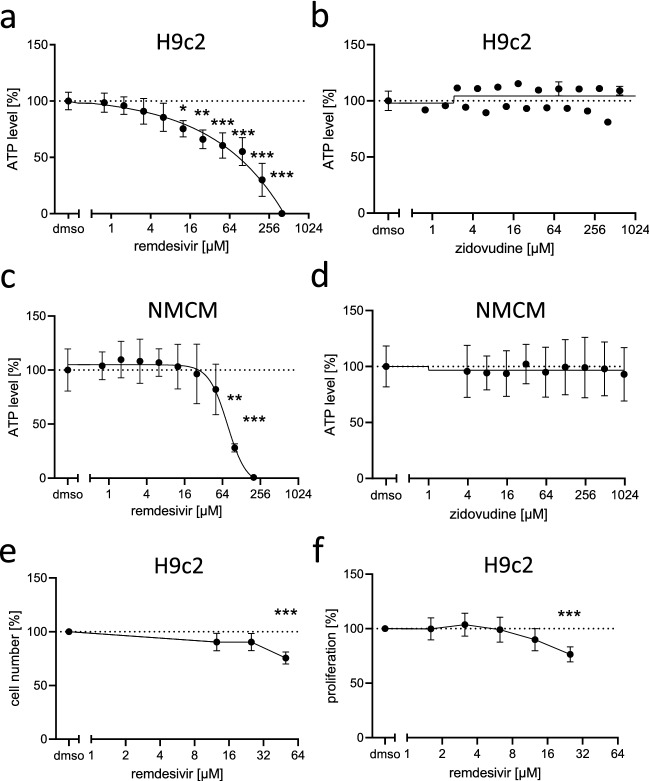
Effect of remdesivir on the viability of NMCM and H9c2 cells. **a**, **b** H9c2 cells or **c**, **d** NMCM were treated with increasing concentrations of remdesivir or zidovudine for 24 h. Viability was determined by measuring ATP levels. Data (*n* = 3, **b**: n =1-2) were normalized to the mean of the solvent control (dmso = 100%; dashed line). Data are presented with a non-linear curve-fit. **e** The relative cell number was determined by nuclei staining 24 h after remdesivir treatment in H9c2 cells (*n* = 6). **f** Proliferation was measured by ^3^H-thymidine incorporation within 6 h after remdesivir treatment in H9c2 cells (*n* = 10). **e**–**f** Data were normalized to the solvent controls (dmso = 100%; dashed line). **a**–**d** Ordinary one-way ANOVA, Dunnett’s multiple comparisons test. **e**–**f** Kruskal–Wallis test with a Dunn’s multiple comparisons test *p < 0.05, **p < 0.01, ***p < 0.001

ATP levels were evaluated as an indicator of cell viability of H9c2 cardiomyoblasts and NMCM after 24 h of remdesivir treatment. Concentrations of up to 400 µM in H9c2 cells and up to 200 µM in NMCM were tested. The respective highest remdesivir concentrations resulted in 100% loss of viability in both cell types as detected by decreased ATP levels, and 12.5 µM in H9c2 cells or 100 µM in NMCM was sufficient to significantly decrease ATP levels (Fig. 1a, c). Even though the nucleoside analog zidovudine is known for mitotoxic effects during HIV therapy and was shown to induce metabolic changes in H9c2 cells at 50 µM after a 6 days treatment (Benbrik et al. 1997; Lund et al. 2007; White 2001), it showed no significant toxicity up to a concentration of 1.2 mM after 24 h in this acute toxicity setup (Fig. 1b, d). Based on these results and considering an assumed concentration range in patients (plasma *C*_max_ = 9 µM), subsequent experiments were conducted at remdesivir test concentrations up to 50 µM.

To analyze, if the decrease in ATP levels was due to a reduction of cell number or of cellular ATP production, the cell number was assessed by nuclei count in response to increasing remdesivir concentrations in the proliferating cardiomyoblast cell line H9c2. This experiment revealed that remdesivir decreases the cell number (12.5 µM; Fig. 1e). Live cell microscopy suggested that the reduced cell number of H9c2 cells treated with remdesivir relative to untreated controls was due to inhibition of cell proliferation rather than induction of cell death (Supp. Videos, Fig. S1a). In line with this, we found no significant release of lactate dehydrogenase (LDH) of remdesivir treated H9c2 cells, which would be indicative of cell death (Fig. S1b). We then measured the proliferative capacity of remdesivir-treated H9c2 cells by ^3^H-thymidine incorporation. Indeed, remdesivir significantly reduced ^3^H-thymidine incorporation in H9c2 cells in a concentration-dependent manner (Fig. 1f). To test whether remdesivir can also impair the growth process in mature non-proliferating cells, we used NMCM. To induce a growth/hypertrophic response in these cells, we applied angiotensin II and IGF and assessed the cardiomyocyte size. However, this process does not seem to be affected by remdesivir (Fig. S7), thus, remdesivir seems to specifically affect cell proliferation or signaling events that may be absent in differentiated cell types.

In conclusion, remdesivir reduces the cell number of the proliferating cardiomyoblast cell line H9c2 by interference with cell proliferation.

### Remdesivir impacts on the beating behavior of neonatal mouse cardiomyocytes

**Fig. 2:**
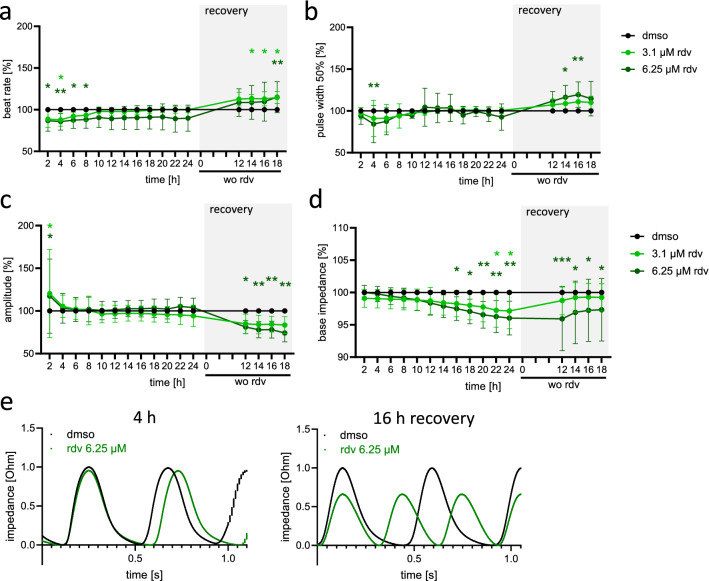
Beating behavior of NMCM is changed by remdesivir. NMCM were treated on day 2 of culture in 1% FCS medium with 3.1 or 6.25 µM of remdesivir (rdv) or with solvent control (dmso). 24 h after treatment remdesivir was removed and medium changed. The parameters beat rate (**a**), pulse width 50% (**b**), beat amplitude (**c**) and base impedance (**d**) were calculated from 20 s impedance recordings every 2 h after treatment. Data were normalized on solvent control (dmso = 100%) in each experiment, *n* = 4–7 independent experiments run with 6 technical replicates (wells) each. Two-way ANOVA, Dunnett’s multiple comparisons test *p < 0.05, **p < 0.01, ***p < 0.001 (**b**: a Grubbs outlier analysis (Alpha = 0.0001) was performed and 3 outliers in rdv 6.25 µM group (6 h: 59.5%, 10 h: 213.9%, 20 h: 440.4%) were removed), **e** representative pulse sequence at 4 h after treatment and 16 h after remdesivir removal

Several case reports and small-scale clinical studies suggest that remdesivir can lead to impaired cardiac function and transient bradycardia (Bistrovic and Lucijanic 2021; Pallotto et al. 2021; Touafchia et al. 2021). To test the direct effect of remdesivir on cardiomyocyte contractility, we treated NMCM that are characterized by spontaneous beating behavior with subtoxic remdesivir concentrations below the *C*_max_ of 9 µM in patients (3.1 or 6.25 µM) for 24 h. The impedance-based Cardio Excyte system (Nanion Technologies) was used to assess the beating behavior and the integrity of the cell monolayer. The beat rate decreased initially by approximately 10%, recovered after 6–10 h and even increased slightly above control levels after remdesivir removal (Fig. 2a). The biphasic effect of remdesivir was also reflected by the pulse width and the amplitude of the beats (Fig. 2b, c, e). The base impedance, a parameter for the integrity of the monolayer, decreased significantly after addition of remdesivir and recovered to some extent after remdesivir washout further supporting the absence of cell death (Fig. 2d). In this experimental setting, the treatment of the cells started 1 day after seeding, when the formation of intercellular connectivity was not fully established. Interestingly, when NMCM were treated at a later time point (day 3 after seeding) the beating behavior was unchanged by remdesivir (Fig. S2). These data suggest that the adverse effects of remdesivir on cardiac function may depend on the current condition of cardiac cells, as, e.g., their metabolic state.

### Remdesivir induces cell type-dependent metabolic changes

**Fig. 3:**
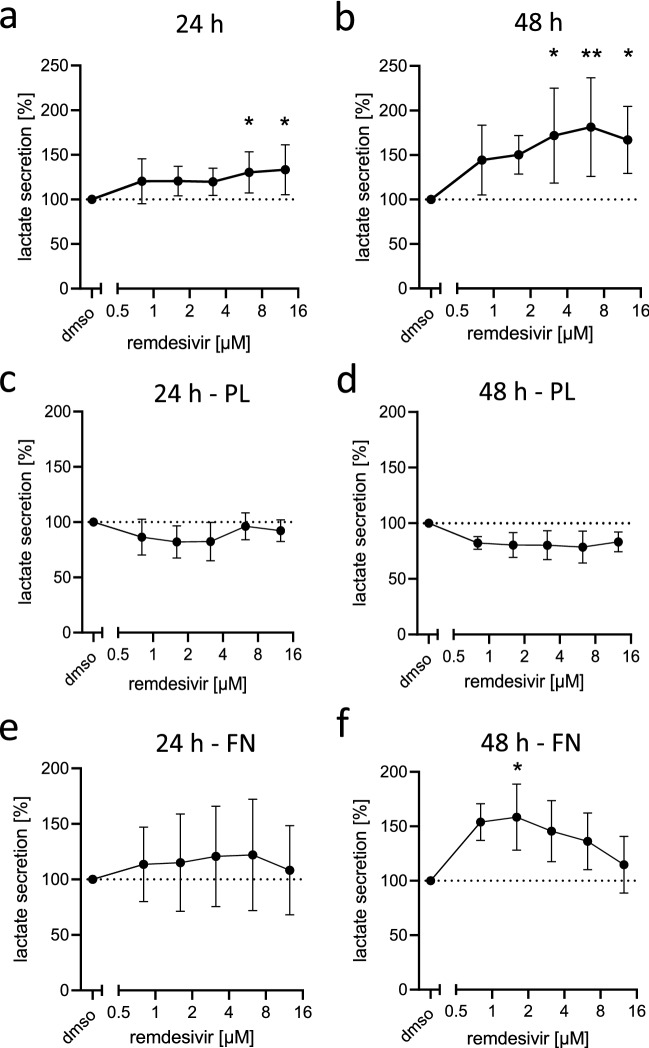
Effect of remdesivir on the metabolism of NMCM and H9c2 cells. **a**, **b** H9c2 cells were treated with indicated concentrations of remdesivir. NMCM were cultured on **c**, **d** poly-lysine (PL) or **e**, **f** fibronectin (FN)-coated 96-well plates for 3 days and subsequently treated with indicated concentrations of remdesivir. After 24 h (**a**, **c**, **e**) or 48 h (**b**, **d**, **f**) lactate concentration was determined in supernatants. Data were normalized to the solvent controls (dmso = 100%; dashed line); *n* = 4–5 independent experiments, Kruskal–Wallis test with Dunn’s multiple comparisons test *p < 0–05, **p < 0.01, ***p < 0.001

Due to the reported mitochondrial toxicity of remdesivir in intestinal and liver cell lines or in isolated mitochondria (Akinci et al. 2020; Fišar et al. 2021), we analyzed its impact on cardiac cells. According to Warburg’s hypothesis, mitochondrial damage is associated with a shift of the cell metabolism from oxidative phosphorylation to aerobic glycolysis and subsequent accumulation and secretion of lactate (Vaupel and Multhoff 2021). In line with reports on mitochondrial toxicity of remdesivir in other cell types, lactate secretion was also induced in a concentration-dependent manner in H9c2 cells after 24 h and even stronger after 48 h (Fig. 3a, b).

**Fig. 4:**
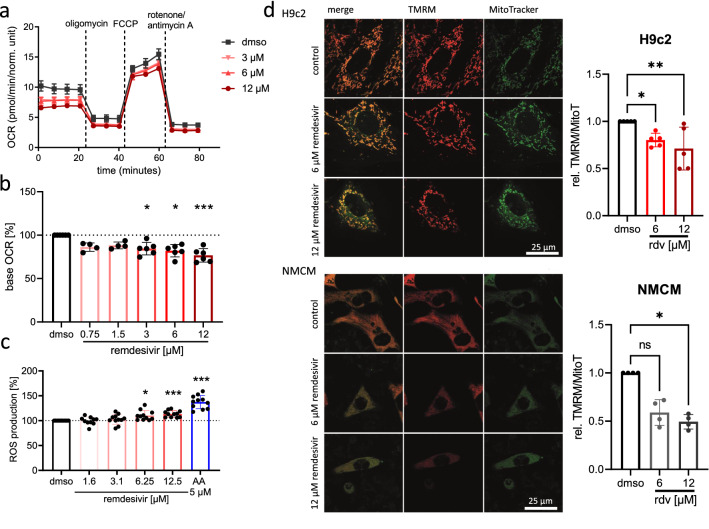
Effect of remdesivir on mitochondrial function in H9c2 cells and NMCM. **a**, **b** H9c2 cells were treated with increasing concentrations of remdesivir for 24 h. 1 h after removal of remdesivir the oxygen consumption rate (OCR) was analyzed in the presence of the inhibitors of the respiratory chain oligomycin, FCCP or rotenone/antimycin A. **a** OCR measurements of one representative experiment (*n* = 2) with H9c2 cells is shown. **b** Base OCR was derived from the difference of the OCR before oligomycin injection and after rotenone/antimycin A (*n* = 4–6). **c** H9c2 cells were treated with increasing concentrations of remdesivir or 5 µM antimycin A (AA) for 24 h. H_2_O_2_ levels were detected in the supernatant by fluorescent labeling (*n* = 7). Data were normalized to the solvent controls (dmso = 100%; dashed line). **d** H9c2 cells (*n* = 5) or NMCM (*n* = 4) were treated for 24 h with remdesivir (rdv) and subsequently mitochondrial membrane potential was measured by microscopic examination. Shown are representative pictures and a quantification of the ratios of TMRM to MitoTracker-signal normalized to the solvent control (dmso = 1). Kruskal–Wallis test with Dunn’s multiple comparisons test *p < 0–05, **p < 0.01, ***p < 0.001

Using the more mature NMCM, we aimed to verify the results seen in H9c2 cells and additionally to test if stressed or resting cardiomyocytes react differently to the mitochondrial toxicity of remdesivir and, therefore, compared its effects on NMCM seeded on poly-lysine (Fig. 3c, d) or fibronectin (Fig. 3e, f) coated cell culture plates. In vitro and in vivo, fibronectin leads to the activation of integrin-receptors and induces cardiomyocyte hypertrophy (Konstandin et al. 2013; Ogawa et al. 2000), while cells are rather inert to poly-lysine (Rainaldi et al. 1998). Indeed, NMCM cultured on fibronectin-coated plates secreted higher levels of lactate compared to those cultured on poly-lysine indicating an impact of the coating on the metabolism of NMCM (Fig. S8c). Interestingly, in NMCM pretriggered by fibronectin, remdesivir (1.6 µM, 48 h) led to lactate secretion (Fig. 4d). Since fibronectin is secreted in cardiac disease (Song et al. 2001; Valiente-Alandi et al. 2018), our results suggest that patients with a pre-existing cardiac damage, may be more susceptible to metabolism-related cardiac side effects of remdesivir.

In conclusion, remdesivir altered the metabolism of H9c2 and NMCM towards glycolysis as shown by lactate secretion.

### Remdesivir induces transient impairment of mitochondrial respiration

Causes for mitochondrial dysfunction are manifold and include modifications of proteins in the electron transport chain, changes in calcium-homeostasis, production of reactive oxygen species, which is often also a consequence of mitochondrial dysfunction, or defects in mitochondrial structure and dynamics (Sun et al. 2021; Zhou and Tian 2018). We used the seahorse technology to characterize the mitochondrial function. We applied this technology to H9c2 cells since this experiment is cell intensive and so far H9c2 cells and NMCM showed a comparable result for the mitochondrial read-out of lactate secretion. Oxygen consumption rate (OCR) was assessed using specific inhibitors of the respiratory chain to allow discrimination between current cell respiration (base OCR), ATP-linked respiration, maximal respiration after decoupling of the proton gradient, and proton-leak. The difference between the base OCR and the maximal respiration is the spare respiratory capacity, which is considered as an indicator of mitochondrial fitness and adaptive capacity to stress conditions (Marchetti et al. 2020). Pretreatment of H9c2 cells with 3 µM or higher concentrations of remdesivir for 24 h decreased the base OCR and the ATP-linked OCR significantly (Fig. 4a, b, Fig. S3a). In addition, the maximal respiration was significantly reduced using 6 or 12 µM of remdesivir (Fig. S3a) and the proton-leak, a process, which consumes oxygen independently of ATP production (Jastroch et al. 2010), was also slightly, but significantly reduced (Fig. S3a). Of note, the unchanged spare respiratory capacity (Fig. S3a), a parameter that is correlated with the enzymatic equipment of the respiratory chain (Desler et al. 2012), indicates that remdesivir did not cause any structural damage to the mitochondria. In line with this, the base OCR recovered within 2–3 h after remdesivir removal (Fig. S4a). To further assess the mitochondrial function, ROS release was evaluated and revealed slightly increased ROS levels in H9c2 cells treated with remdesivir for 24 h (6.25 and 12.5 µM; Fig. 4c). To further validate the findings and to evaluate the transferability of the results to primary cardiomyocytes, the stability of the mitochondrial membrane potential in H9c2 cells and NMCM after a 24-h treatment with remdesivir was assessed, a parameter that correlates well with mitochondrial defects (Amacher 2005; Zorova et al. 2018). In both cardiac cell types, remdesivir (6–12 µM) resulted in a significant collapse of the mitochondrial membrane potential (Fig. 4d).

We conclude that a 24-h treatment of remdesivir lowers the respiratory activity and mitochondrial membrane potential of H9c2 cells and NMCM, but does not seem to induce an irreversible mitochondrial damage.

### cAMP analogs lead to a partial rescue of mitochondrial dysfunction, but not cell viability

**Fig. 5:**
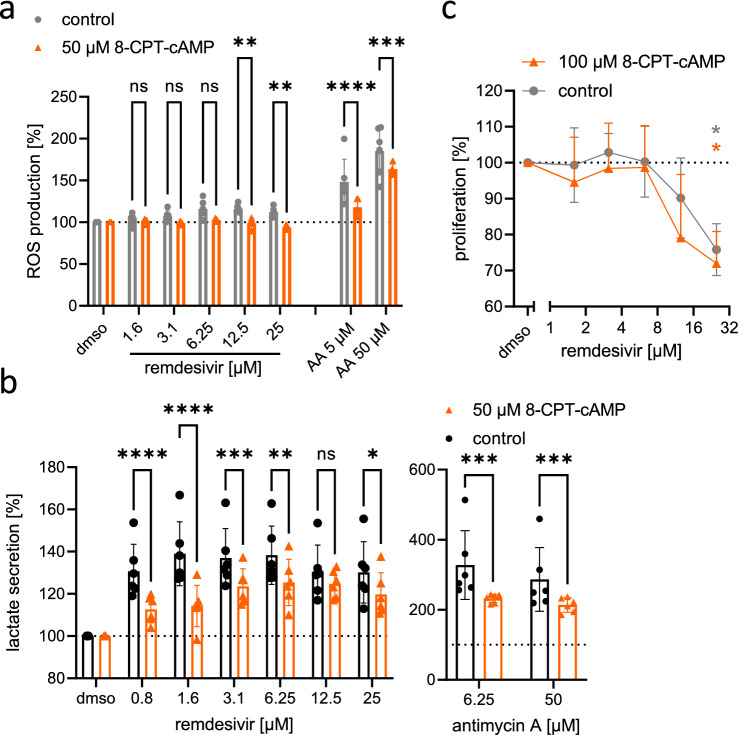
The effect of cAMP on the toxicity of remdesivir. **a** H9c2 cells were treated with increasing concentrations of remdesivir or antimycin A in the presence or absence of 50 µM of 8-CPT-cAMP for 24 h. H_2_O_2_ levels were detected in the supernatant by fluorescent labeling (*n* = 6). **b** H9c2 cells were treated with increasing concentrations of remdesivir or antimycin A in the presence or absence of 50 µM 8-CPT-cAMP for 48 h. Lactate concentration was measured in the supernatant (*n* = 6). **c** Proliferation of H9c2 (n = 5; *control values in grey also included in *Fig. 2b) was measured by ^3^H-thymidine incorporation within 6 h after remdesivir treatment in the presence or absence of 100 µM 8-CPT-cAMP. Data were normalized to the solvent controls (dmso = 100%; dashed line). 2-way ANOVA with Sidak’s multiple comparisons test (**a**, **b**) or Kruskal–Wallis test with Dunn’s multiple comparisons test (**c**). *p < 0.05, **p < 0.01, ***p < 0.001

Even though mitochondria were affected by remdesivir treatment, in none of our cell models changes in mitochondrial DNA content were detected, a hallmark of nucleoside analog toxicity (Young 2017) (Fig. S5a–e). To further understand how remdesivir impacts on mitochondria in cardiomyocytes, we hypothesized that remdesivir inhibits adenylyl cyclase and subsequently reduces cAMP levels, since the ATP binding pocket of adenylyl cyclase and the one of polymerases have significant similarities (Tesmer and Sprang 1998). Such a cAMP-dependent effect on complex I phosphorylation of the respiratory chain has been reported for zidovudine (Lund and Wallace 2008). To test this hypothesis, we assessed the effect of remdesivir on ROS- and lactate production in H9c2 cells in the presence or absence of an excess of cell-permeable cAMP-analog 8-CPT-cAMP (50 µM). Since mitochondrial sensitivity to remdesivir was comparable for H9c2 and NMCM, we used H9c2 cells for this experiment. 8-CPT-cAMP partially rescued the observed adverse effects of remdesivir such as ROS production and lactate secretion (Fig. 5a, b). Similarly, 8-CPT-cAMP partially rescued the mitotoxic effects of antimycin A (Fig. 5a, b). Of note, the 8-CPT-cAMP did not ameliorate the adverse effect of remdesivir on H9c2 cell proliferation (Fig. 5c). This indicates that the metabolic changes induced by remdesivir were not causal for its effect on proliferation. In line with this, also the reduction of ATP levels from H9c2 cells after remdesivir treatment was unaltered, when cells were dependent on mitochondrial respiration for energy production due to galactose as energy source (Fig. S6a).

In conclusion, altered cAMP homeostasis may be one aggravating factor for the mitochondrial toxicity of remdesivir, but mitochondrial toxicity is not causal for impaired cell proliferation.

### Remdesivir has cell type-dependent effects on proliferation and metabolism of kidney cells

**Fig. 6:**
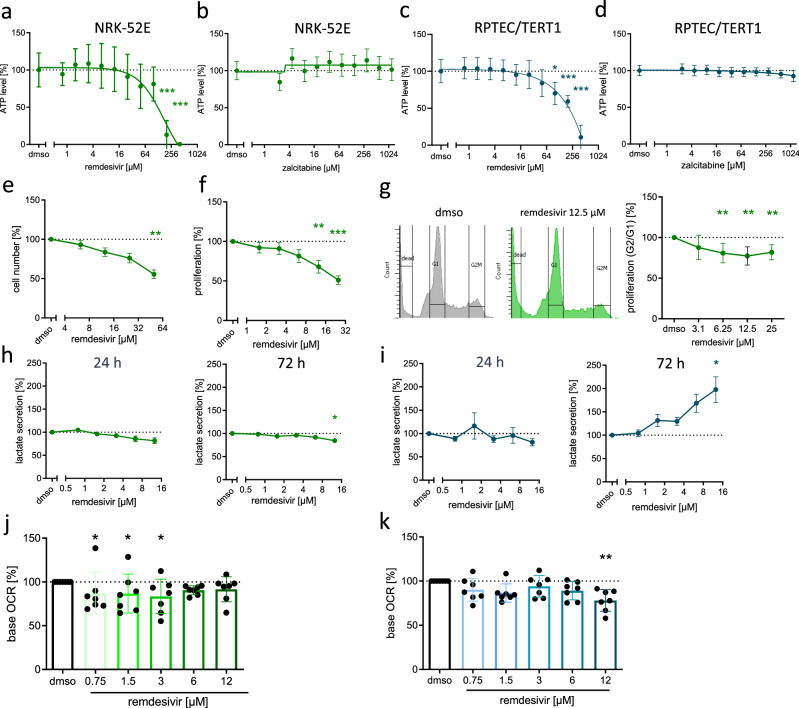
Effect of remdesivir on the viability of kidney cells. **a**, **b** NRK-52E cells (*n* = 6–7) or **c**, **d** RPTEC/TERT1 (*n* = 5) were treated with increasing concentrations of remdesivir or zalcitabine for 24 h. Viability was determined by measuring ATP levels. Data were normalized to the mean of the solvent control (dmso = 100%; dashed line). Data are presented with a non-linear curve-fit. Ordinary one-way ANOVA, Dunnett’s multiple comparisons test. **e** The relative cell number was determined by nuclei staining 24 h after remdesivir treatment in NRK-52E cells (*n* = 8–9). **f**, **g** Proliferation of NRK-52E cells was measured by **f** ^3^H-thymidine incorporation within 6 h after remdesivir treatment (*n* = 5) and **g** by cell cycle analysis (*n* = 7). **h** NRK-52E (*n* = 5–9) or **i** RPTEC/TERT1 (*n* = 6–9) cells were treated with increasing concentrations of remdesivir for indicated time periods. Lactate concentration was measured in supernatants. **j** NRK-52E or **k** RPTEC/TERT1 cells were treated with increasing concentrations of remdesivir for 24 h. 1 h after removal of remdesivir the oxygen consumption rate (OCR) was in the presence of the inhibitors of the respiratory chain oligomycin, FCCP or rotenone/antimycin A. Base OCR was derived from the difference of the OCR before oligomycin injection and after rotenone/antimycin A (*n* = 5–6). *p < 0.05, **p < 0.01, ***p < 0.001

To evaluate the toxicity of remdesivir on kidney cells, we used normal rat kidney cells (NRK-52E) and RPTEC/TERT1. ATP levels were reduced with increasing concentrations of remdesivir (24 h) in both cell lines and reached significance at a concentration of 200 µM in NRK-52E and 100 µM in RPTEC/TERT1 cells (Fig. 6a, c). For comparison, the nucleoside analog zalcitabine that reduced the mitochondrial DNA content in RPTEC/TERT1 cells (Fig. S5e) had no effect on the viability of either of the cell lines at concentrations up to 1.2 mM (Fig. 6b, d). Of note, ATP levels in RPTEC/TERT1 cells decreased already at lower remdesivir concentrations after longer treatment (120 h; Fig. S6b) indicating a delayed response of RPTEC/TERT1 cells to remdesivir treatment. Since we observed a higher basal proliferation rate in NRK-52E cells, we used these cells for the evaluation of the impact of remdesivir on cell number and proliferation. The cell number was significantly reduced after a 24-h treatment with 50 µM remdesivir (Fig. 6e) and proliferation as measured by ^3^H-thymidine incorporation was significantly reduced by even lower remdesivir concentrations (12.5 and 25 µM; Fig. 6f). We further applied a cell cycle analysis to validate an anti-proliferative effect of remdesivir in these cells. Indeed, the proportion of cells in the G2/M phase, which represents dividing cells, was significantly lower in remdesivir-treated cells (Fig. 6 g). Of note, we did not detect remdesivir-induced cell death in NRK-52E cells as measured by LDH-release or increased dead cell fractions in FACS analyses (Fig. S1c–e). Thus, these results indicate that remdesivir impairs the proliferation but not the viability of NRK-52E cells similarly as observed in H9c2 cells.

Potential changes in cell metabolism of both kidney cell lines were analyzed by lactate secretion, that did not reveal alterations of remdesivir treatment up to 24 h (Fig. 6h, i). However, after 72 h, there was a significant increase of lactate secretion in RPTEC/TERT1 (Fig. 6i), suggesting a delay in the mitotoxic response. Further, remdesivir reduced base OCR and ATP-linked respiration of RPTEC/TERT1 cells at high concentrations (Fig. 6k, Fig. S3c); however, the effects appeared to be reversible after remdesivir wash-out (Fig. S4b, c). In contrast to RPTEC/TERT1, that have a relatively high mitochondrial content and are thought to strongly depend on the mitochondrial integrity/functionality, remdesivir did not induce lactate secretion in NRK-52E cells. In addition, no clear reduction of the base OCR or other parameters of mitochondrial respiration were observed in NRK-52E cells and ROS production only at a very high remdesivir concentration (Fig. 6j; Fig. S8b). Similarly as seen for H9c2 cells, the energy loss due to mitotoxicity was not the main cause for reduced ATP levels as shown by similar effects of remdesivir when RPTEC/TERT1 and NRK-52E cells were dependent on mitochondrial respiration for energy production due to galactose as energy source (Fig. S6b, c).

In conclusion, remdesivir impaired the proliferation of NRK-52E cells with low mitochondrial content and impaired the mitochondrial activity in RPTEC/TERT1 cells in a reversible manner, which did not impact on the amount of viable cells.

### Remdesivir induced changes in the proteome

To get further insights into the underlying processes leading to the adverse effects of remdesivir, we performed mass spectrometry analyses of NMCM and RPTEC/TERT1 as these cells represent the more mature cardiac- and kidney cell types (Han et al. 2020; Simon et al. 2014; Watkins et al. 2011; Wieser et al. 2008). For these experiments, we used a remdesivir concentration corresponding to the *C*_max_ in patients (9 µM) and a subtoxic concentration of antimycin A (0.2 µM) to assess proteomic changes due to a blockage of mitochondrial respiration. In total, 2875 (NMCM) and 3801 (RPTEC/TERT1) proteins were quantified. In remdesivir-treated NMCM, 39 proteins were differentially regulated (adj. *p* < 0.05, fold-change > 2), 22 downregulated and 17 upregulated. Among those proteins that were regulated in response to remdesivir 16 proteins were regulated to a similar extent in response to antimycin A suggesting that remdesivir impacts at least to some extent on similar proteins as the mitotoxic antimycin A (Table S2). In remdesivir-treated RPTEC/TERT1 cells, 36 proteins were differentially regulated (adj. *p* < 0.05, fold-change > 2), 20 downregulated and 16 upregulated. Among those proteins that were regulated in response to remdesivir, 17 proteins were regulated to a similar extent in response to antimycin A (Table S3). To assess the pathways affected by remdesivir, we applied gene-ontology based analyses using the Database for Annotation, Visualisation and Integrated Discovery (DAVID) (Huang da et al. 2009) and included proteins that were significantly regulated (adj. *p* < 0.05) with a fold-change > 1.5 in NMCM and RPTEC/TERT1 (95 proteins in NMCM and 113 proteins in RPTEC/TERT1 cells. Considering upregulated proteins in NMCM there was no significantly enriched GO term in the sections biological process and molecular function. Several GO terms in the section cellular compartments were significantly affected: the GO terms “membrane” (adj. *p* = 0.021) and “respiratory chain” (adj. *p* = 0.024). The “respiratory chain” was identified as part of a cluster (enrichment score 2.6) including the GO terms “mitochondrion” (adj. *p* = 0.21) and “mitochondrial inner membrane” (adj. *p* = 0.21). Upregulated proteins of the respiratory chain were NADH dehydrogenase subunit 1 (ND1; 1.9-fold), ND4 (1.44-fold), ND5 (1.33-fold) and cytochrome c oxidase subunit 1 (COX1; 1.47-fold), which were all also upregulated by antimycin A. Considering the downregulated proteins in NMCM the GO terms “intermediate filament” (adj. *p* = 0.001) and “keratin filament” (adj. *p* = 0.007) were most significantly enriched (Table 1). This was due to downregulation of keratin 2 (Krt2; 0.31-fold), Krt1 (0.36-fold), Krt10 (0.46-fold), Krt5 (0.49-fold), Krt14 (0.61-fold), Krt42 (0.66-fold). Except for Krt14 and Krt42, similar changes were identified in antimycin A treated cells. In RPTEC/TERT1 cells, the only significant GO term was “protein binding” (adj. *p* = 0.042) in the section molecular function among the downregulated proteins (Table 1) indicating a lower sensitivity of RPTEC/TERT1 cells compared to NMCM. Thus, at this early time point after treatment, no downregulation of mitochondrial proteins was detected and mitochondrial toxicity of remdesivir was, if at all, only indirectly represented in the proteome.

**Table 1:** GO-ontology based analysis of differentially regulated proteins with DAVID

Downregulated (adj. *p* value)			Upregulated (adj. *p* value)		
MF	BP	CC	MF	BP	CC
*NMCM *					
		Intermediate filament (0.001)			**Respiratory chain (0.024)**
ns	ns	Keratin filament (0.007)	ns	ns	Membrane (0.021)
		Extracellular exosome (0.025)			Extracellular exosome (0.037)
*RPTEC/TERT1 *					
ns	ns	ns	Protein binding (0.042)	ns	ns

Proteins > 1.5-fold differentially regulated by 12 h treatment with 9 µM remdesivir (adj. P < 0.05) were selected (95 proteins in NMCM; 112 proteins in RPTEC/TERT1) and subjected to GO-ontology based analysis in DAVID

*MF* molecular function, *BP* biological process, *CC* cellular compartment, *adj p value* Benjamin–Hochberg-adjusted *p* value, *ns* not significant, *bold letters* part of cluster; 96–100% of selected proteins were included in the analysis

## Discussion

With respect to current cardiac and kidney safety concerns due to remdesivir treatment (Abdelmajid et al. 2021; Chouchana et al. 2021; Gérard et al. 2021; Pallotto et al. 2021; Rafaniello et al. 2021; Touafchia et al. 2021), we here investigated the toxicity of remdesivir in cardiac and kidney cells and found adverse effects of remdesivir on cell proliferation, mitochondrial function, and the beating behavior of NMCM.

The effects of remdesivir on cell viability are highly diverse. The differential impact of remdesivir could be due to different treatment periods (Akinci et al. 2020; Choi et al. 2020; Xu et al. 2020), cell types and experimental readouts. In particular, the expression patterns/levels of cellular transporters for remdesivir or its metabolites may alter among cell types and thus their sensitivity for remdesivir-induced adverse effects (Akinci et al. 2020; Ambrus et al. 2021; Miller et al. 2021; Nies et al. 2021; Telbisz et al. 2021). For example, Xu et al. (2020) tested several cell types and the only cell line with clear mitochondrial toxicity (PC-3) was the one with the highest intracellular amount of remdesivir triphosphate (RTP), the active metabolite of remdesivir. However, the transporters for remdesivir or its metabolites are not entirely known. Since the cell types used in our study differ in species, differentiation state and tissue type, and possess different metabolic and functional properties, they can cover the cellular diversity and thus the impact of remdesivir toxicity especially in cardiac and kidney cells to a reasonable extent. Even though we detected a lower cell number in the proliferating cells (NRK-52E, RPTEC/TERT1 and H9c2 cells), we identified anti-proliferative effects of remdesivir as the cause for these observations. While remdesivir reduced the proliferative activity after 6 h of treatment in NRK-52E cells and H9c2 cells, which correlated well with a reduced ATP signal and cell number after 24–72 h, similar remdesivir-mediated effects on the ATP signal in RPTEC/TERT1 cells needed incubation times of about 120 h (Fig. S4b). This postponed effect of remdesivir on the ATP levels in RPTEC/TERT1 cells is most likely due to a slower replication rate of RPTEC/TERT1 cells and further validates our conclusion that remdesivir attenuates cell proliferation rates. Of note, remdesivir had no significant effect on hypertrophic growth in the non-proliferating cell type, i.e., NMCM. Thus, the anti-proliferative effect of remdesivir seems to impact only on proliferating cell types, but this could be of great importance in certain disease conditions, e.g., it may delay wound healing in organs with necrotic damage, e.g., the kidney or the heart, where for example fibroblasts, endothelial or immune cells are involved in organ remodeling (Desmoulière et al. 2003).

Molecularly, the impact of remdesivir on cell proliferation is still unclear. The impaired proliferation could possibly be due to inhibition of DNA-polymerases, a phenomenon critically to be obviated during the design and development of antiviral nucleotide-analogs (Schaich et al. 2017), however, no interactions of remdesivir with DNA-polymerases α or β were identified so far (Xu et al. 2020). Alternatively, direct or indirect effects of remdesivir on intracellular signaling pathways or gene expression may contribute to the observed effects on proliferation.

With respect to the assessment of the impact of remdesivir on cell metabolism, we employed widely accepted methods in the in vitro assessment of mitochondrial toxicity in the cell types that have high contents of mitochondria and that metabolically are highly dependent on intact mitochondrial function, i.e., H9c2, NMCM and RPTEC/TERT1 (Hynes et al. 2013; Rana et al. 2011; van der Stel et al. 2020). There was a clear mitotoxic effect in H9c2 cells, RPTEC/TERT1 cells and NMCM as measured by lactate secretion, oxygen consumption and/or collapse of the mitochondrial membrane potential. The concentration–response analyses revealed different sensitivities for the respective cell types, which might most likely be due to different metabolic plasticity of the cells. For example, NMCM are less capable to switch from oxidative phosphorylation to glycolysis compared to H9c2 cells and thus lactate secretion was already significantly reduced under basal/unstressed conditions (Fig. S8c). In addition, RPTEC cells were less sensitive, most likely due to their strong drug metabolizing activity and relevant expression levels of outward drug transporters (Hall et al. 2021), which may reduce the exposure to remdesivir and, therefore, cause the observed delay of the mitotoxic responses to remdesivir.

NRK-52E cells hardly depend on the energy production of mitochondria as envisioned by their inertness to mitotoxicant antimycin A that did not lead to an increase of lactate secretion in NRK-52E cells (Fig. S9a). However, mitochondria in NRK-52E cells were not inert to remdesivir as ROS release was detected after treatment with antimycin A or a high dose of remdesivir (50 µM; Fig. S9b). Of interest, in line with the low mitochondrial content of NRK-52E compared to H9c2 cells (Kuznetsov et al. 2015), ROS was detected after application of 50 µM of remdesivir in NRK-52E cells while 6.25 µM were sufficient for ROS detection in H9c2 cells (Fig. S9b and Fig. 4c).

Seahorse analysis revealed that the spare respiratory capacity, representing a parameter for structural mitochondrial damage (Marchetti et al. 2020) was unchanged, which can explain the reversibility of the mitochondrial phenotype after remdesivir removal observed in H9c2 and RPTEC/TERT1 cells in the seahorse analysis for oxygen consumption rate (Fig. S4). Though, it cannot be excluded that a prolonged treatment of the cells with remdesivir would proceed the mitochondrial phenotype to irreversible damage as it was shown in studies with PC-3 cells (Xu et al. 2020) and in human induced pluripotent stem cell-derived cardiomyocytes (Kwok et al. 2021).

Of note, an altered energy metabolism was not causal for the effects on cell number of remdesivir, as the cell number in cells, which were forced to retrieve their energy via mitochondrial respiration by the use of galactose-containing medium was similarly affected by remdesivir compared to cells in glucose-containing medium, which can switch to glycolysis to cover their energy demand. Therefore, several distinct molecular initiating events caused by remdesivir may cause the different observed adverse effects.

Molecularly, remdesivir has been reported to attenuate the respiratory chain complex I and complex II activity similarly as other nucleoside analogs (Fišar et al. 2021; Lund and Wallace 2008). In addition, a decrease in mitochondrial respiratory gene expression has been suggested by RNA-seq gene expression clustering, as nuclear genome-encoded proteins of complex I, II, IV and V were affected in HepG2 cells after a 24-h treatment with remdesivir (Akinci et al. 2020). Of interest, we observed remdesivir-induced induction of the proton-leak in H9c2 cells, defined as the oxygen consumption in the presence of the ATP-synthase-inhibitor oligomycin (Fig. S3a). A similar effect has been demonstrated for the nucleoside analog zidovudine in rat heart mitochondria (Valenti et al. 2000). A physiological role for the proton-leak, that is likely to occur, may be maintaining carbon flux despite low ATP demand or prevention of ROS production according to the “uncoupling to survive” hypothesis (Jastroch et al. 2010). Future studies will have to evaluate the significance of remdesivir-mediated inhibition of the proton-leak for the overall mitochondrial function and pathophysiological conditions. For example, mitochondrial dysfunction is a frequent consequence of HIV therapy with nucleoside analogs and elevated lactate levels are a common side effect, which can lead to lactic acidosis with symptoms such as nausea, vomiting, loss of weight, gradual numbness of the legs, abdominal pain, and heavy breathing (Chagoma et al. 2013). It remains of course to be determined, if the duration and dosing of remdesivir treatment is sufficient to induce a situation of lactic acidosis in humans, which can be addressed therapeutically (Kraut and Madias 2016). Prolonged reduction of the mitochondrial membrane potential might also lead to a prolonged energy deprivation and thereby to secondary persistent cell damage and respective consequences for organ function and remodeling.

Regarding the herein observed transient reduction of beat rate of NMCM after remdesivir treatment at concentrations lower than the *C*_max_ is in line with reports of reversible bradycardia in patients (Bistrovic and Lucijanic 2021; Pallotto et al. 2021; Touafchia et al. 2021). In addition, two studies using human stem-cell-derived cardiomyocytes for electrophysiological analyses identified similar effects of remdesivir on cell contractility, such as changes in field potential duration, reduction of spontaneous beating rates and changes of ion currents (Rossello et al. 2019; Yanagida et al. 2021). Our data show in addition to a remdesivir mediated early transient decrease of the beat rate and a delayed reduction of the base impedance, an indicator for cell monolayer integrity (16 h after treatment), a rebound effect after drug removal, as indicated by an increased beat rate and by a decreased beating force (amplitude; Fig. 3). Since treatments at a later time point in culture, i.e., a presumably less vulnerable time point with more stably connected cardiomyocytes, did not alter the beat rate (Fig. S2) and since only stressed fibronectin-stimulated NMCM revealed increased lactate production after remdesivir treatment (Fig. 3), the overall disease status of the cardiac tissue may determine the grade of remdesivirs’ cardiotoxicity.

The use of human stem cell-derived cardiomyocytes for the identification of arrhythmogenic properties of drugs is a central element of the CiPA initiative (Gintant et al. 2016). In addition, it was claimed that inclusion of structural toxicity, which might occur with some delay after drug exposure with subsequent impact on contractility, should be included in in vitro approaches (Yang and Papoian 2018). The delayed changes of beat rate, impedance and beat amplitude observed in NMCM in our study (Fig. 3) may be an indicator for structural toxicity of remdesivir. Of note, a 3-day treatment with remdesivir by Kwok et al. showed a reduction of mitochondrial respiration and altered electrophysiological properties towards a slower beating frequency of hiPSC-CM, which was associated with increased fission and irreversible disorganization of mitochondria (Kwok et al. 2021). Therefore, we consider the Nanion Cardio Excyte as an appropriate tool to discriminate between transient and permanent structural damage. Even though we did not assess, if mitochondrial toxicity was causal for functional impact of remdesivir on NMCM, our data on the contractile behavior of NMCM after remdesivir treatment, indicate early, late and compensatory effects on cell beating. Therefore, also because of the impact of remdesivir on cardiomyocyte beat rate, a thorough surveillance of cardiac function during and after the therapy should be provided.

We aimed to get insights into early molecular initiating events of remdesivir on the proteome in NMCM and RPTEC/TERT1 cells. These cells were chosen since they are largely resembling the maturated characteristics of primary cardiomyocytes and renal proximal tubular cells in terms of protein expression, mitochondrial content and functionality (Han et al. 2020; Simon et al. 2014; Watkins et al. 2011; Wieser et al. 2008). Of interest, almost half of the differentially regulated proteins by remdesivir in both cell types were also regulated by antimycin A treatment, indicating a certain similarity of remdesivirs’ toxicity to the mitochondrial toxicity of antimycin A. The GO terms “intermediate filament” and “keratin filament” that were significantly affected by remdesivir treatment in NMCM might be the first indication for the initiation of mitochondrial disorganization, a process described in Kwok et al., since keratins have been shown to reorganize mitochondria in keratinocytes (Steen et al. 2020). Of note, ectopic expression of other keratins (K8 and K18) in cardiomyocytes is associated with failing human myocardium (Papathanasiou et al. 2015).

The proteomic data presented for the NMCM are supportive for the observed mitochondrial toxicity of long-term treatment with remdesivir as observed by other groups (Kwok et al. 2021). A reason for the absence of evidence in proteomic data for mitotoxicity in RPTEC/TERT1 cells might be a more extensive metabolization and cellular export of remdesivir in RPTEC/TERT1 cells, for which a delayed response to remdesivir was also observed in the other endpoints (e.g., reduced amounts of viable cells, increased lactate secretion). The presented data will be important for future mechanistic research about remdesivir toxicity and also toxicity of similar nucleoside analogs.

In our study, we assessed the toxicity for remdesivir in vitro using different types of cardiac and kidney cells as model systems. Our analyses revealed an adverse effect of remdesivir on the amounts of viable cells due to impaired cell proliferation and energy metabolism via mitochondrial toxicity as evidenced by increased lactate secretion and reduced oxygen consumption rates. Functionally, the anti-proliferative effect of remdesivir might be of interest in situations when tissue renewal/repair is of need; its impact on energy metabolism particularly in cardiomyocytes that depend on mitochondria as energy source might subsequently impact on the heart’s contractile function and rhythm. Impedance analyses of beating cardiomyocytes mirrored the bradycardia observed in patients after remdesivir application.

In the light of upcoming approaches to orally deliver the remdesivir nucleoside GS-441524 or other pro-drugs with higher oral bioavailability (Li et al. 2021; Lo et al. 2021; Schäfer et al. 2022, 2021; Xie and Wang 2021) or to administrate remdesivir in earlier stages of COVID-19 to elevate its antiviral efficacy (EMA 2021a; FDA 2022; Gottlieb et al. 2022; Paranjape et al. 2021; Razzack et al. 2021), our results argue for a careful surveillance of cardiac function during therapy.

## Supplementary Information

Below is the link to the electronic supplementary material.

Supplementary file1 (DOCX 212 kb)

Supplementary file2 (PDF 929 kb)

Supplementary file3 (7Z 175910 kb)

## Data Availability

The mass spectrometry proteomics data have been deposited to the ProteomeXchange Consortium via the PRIDE (Perez-Riverol et al. 2019) partner repository with the dataset identifier PXD029311.
