# Supplementary material for: The potential of remdesivir to affect function, metabolism and proliferation of cardiac and kidney cells in vitro
Source: Arch Toxicol. 2022 May 17;96(8):2341–60. doi: 10.1007/s00204-022-03306-1 (PMC9110936; doi:10.1007/s00204-022-03306-1)
Supplement: Supplementary file 1 — Supplementary file1 (DOCX 212 kb) [file 204_2022_3306_MOESM1_ESM.docx]

**The potential of remdesivir to affect function, metabolism and proliferation of cardiac and kidney cells in vitro**

Katja Merches^1,2^, Leonie Breunig^1^, Julia Fender^1^, Theresa Brand^1^, Vanessa Bätz^1^, Svenja Idel^3^, Laxmikanth Kollipara^3^, Yvonne Reinders^3^, Albert Sickmann^3,4,5^, Angela Mally^1^, Kristina Lorenz^1,3,6^

^1^ Institute of Pharmacology and Toxicology, University of Würzburg, Würzburg, Germany

^2^ current address: Landesamt für Gesundheit und Lebensmittelsicherheit (LGL), Erlangen, Germany

^3^ Leibniz-Institut für Analytische Wissenschaften – ISAS – e.V., Dortmund, Germany

^4^ Department of Chemistry, College of Physical Sciences, University of Aberdeen, Aberdeen, Scotland, United Kingdom

^5^ Medizinische Fakultät, Medizinisches Proteom-Center (MPC), Ruhr-Universität Bochum, Bochum, Germany

^6^ PGS Toxicology and Environmental Protection, University of Leipzig, Johannisallee 28, Leipzig, Germany

Corresponding author:

Prof. Dr. Kristina Lorenz, [lorenz@toxi.uni-wuerzburg.de](mailto:lorenz@toxi.uni-wuerzburg.de)

**Supplementary Information**

**Supplementary Methods**

*Cell models*

H9c2 cells are cardiomyoblasts originally isolated from a rat heart (Kimes and Brandt 1976) and have often been used as cell model to assess pathways responsible for heart failure and cardiotoxic properties of chemicals or drugs (Bouitbir et al. 2022; Li et al. 2021; Miyoshi et al. 2022; Wang et al. 2022). Different cell lines have been compared to primary cardiomyocytes and H9c2 cells were found to have a reasonable similarity with regards to energy metabolism compared to other available cardiomyoblast cell lines such as HL-1 cells (Kuznetsov et al. 2015). Furthermore, H9c2-cells and neonatal cardiomyocytes have been reported to respond similarily to hypertrophic stimuli in vitro (Han et al. 2020; Watkins et al. 2011).

Neonatal mouse cardiomyocytes have a long tradition for disease related studies and cardiotoxic effects. The heart beat of mice in vivo is about 10 times faster than in humans (Janssen et al. 2016). In vitro, the NMCM beat rate is 60-100 bpm, thus in the range of human tissue (Webster and Patrick 2000). Shortly after birth, mouse cardiomyocytes switch from proliferation to hypertrophic growth, which comes along with a metabolic shift towards fatty acid oxidation (Lalowski et al. 2018). Therefore, we considered them as an appropriate in vitro model for the toxicity-assessment of a presumably mitotoxic drug.

NRK-52E cells are an EGF-responsive epithelioid clone of the normal rat kidney cell line, which was cultivated from whole kidney suspensions from young and healthy Osborne-Mendel rats (de Larco and Todaro 1978; Huu et al. 1966). NRK-52E cells differ in the expression of tight-junction proteins, proximal tubulular marker-proteins and characteristic transporter proteins from endogenous rat renal tubular epithelial cells and should therefore be regarded as an immature cell type (Lechner 2014). NRK-52E cells possess less mitochondria than rat renal proximal tubules in vivo and are less sensitive to cytotoxic stimuli compared to primary cultures but have been established as a valuable tool to detect cytotoxic effects relevant for renal injury (Lash et al. 2002).

RPTEC/TERT1 cells are renal proximal tubule epithelial cells of a healthy human donor, which were immortalized using the human telomerase reverse transcriptase (hTERT) subunit (Wieser et al. 2008). They show characteristic morphological and functional properties and have been identified as a useful model for toxicity studies on cadmium and benzo(a)pyrene (Simon et al. 2014).

H9c2 cells and NRK-52E cell lines are immature proliferative cells with a limited potential to predict specific cardio- or nephrotoxicity (Lin and Will 2012). However, we included these cell types as animal-free and well characterized cell lines that have often been used to assess cardiotoxicity of nuleoside-analogs and cells that will therefore help to increase our mechanistic understanding of remdesivir toxicity (Liu et al. 2012; Liu et al. 2015; Liu et al. 2014; Lund et al. 2007; Lund and Wallace 2004; Lynx et al. 2008).

*LDH-assay for detection of cell-damage*

Secreted lactate dehydrogenase (LDH) from damaged cells was measured in supernatant of cultured cells by a colorimetric assay, in which LDH converts lactate into pyruvate under the consumption of beta-nicotinamide adenine dinucleotide sodium salt (NAD, Sigma) according to the protocol of Kaja et *al*. (Kaja et al. 2015). The ingredients of the color solution were 1-methoxyphenazine methosulfate (MPMS), iodonitrotetrazolium chloride (INT), lithium-L-lactate and Tris base were purchased from Sigma. Shortly, 50 µl of supernatant was mixed with 50 µl of color-solution, incubated for 5 min before stopping of the reaction with 1 M acetic acid (Sigma). Absorption was detected at 490 nm with the Synergy Neo2 multi-mode reader (BioTek) and the software Gen5™ 3.10 (BioTek). Treatment with 10 - 20 % dimethyl sulfoxide (DMSO; Sigma) or 50 µM antimycin A (Sigma), was used as a positive control and culture medium as a negative control. The relative cytotoxicity [%] was calculated as follows:


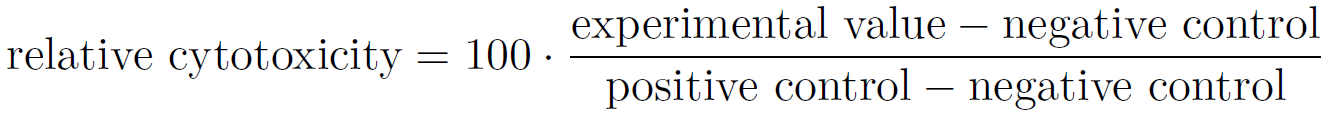


*Proteomics*

***Materials***

Following chemicals were purchased from Sigma-Aldrich, Germany: ammonium bicarbonate (NH_4_HCO_3_) anhydrous magnesium chloride (MgCl_2_), iodoacetamide (IAA) complete mini EDTA-free protease inhibitor cocktail tablets and triethylammonium bicarbonate (TEAB). Tris base was bought from Applichem Biochemica, Darmstadt, Germany. Sodium dodecyl sulfate (SDS) was purchased from Carl Roth, Karlsruhe, Germany. Dithiothreitol (DTT) was bought from Roche Diagnostics, Mannheim, Germany. Sodium chloride (NaCl) was purchased from Merck, Darmstadt. Benzonase Nuclease was purchased from Novagen. Sequencing grade modified trypsin was purchased from Promega, Madison, USA. Bicinchoninic acid assay (BCA) kit was bought from Pierce Thermo Fisher Scientific, Schwerte, Germany. S-Trap spin columns were purchased from ProtiFi LLC, Farmingdale NY 11735, USA. All chemicals for ultra-pure HPLC solvents such as formic acid (FA), trifluoroacetic acid (TFA) and acetonitrile (ACN) were purchased from Biosolve, Valkenswaard, the Netherlands.

***Cell lysis, carbamidomethylation and S-Trap on-filter proteolysis***

Neonatal mouse cardiomyocytes (NMCM) and human renal proximal tube epithelial cells (RPTEC) that were treated with either DMSO (control), remdesivir [9 µM] or antimycin A [0.2 µM] were lysed with 1% SDS buffer containing 50 mM Tris-HCl, 150 mM NaCl, pH 7.8 with cOmplete^TM^ Mini (Sigma). To degrade nucleic acids, cell lysates were treated with Benzonase plus 2 mM MgCl_2_ and incubated at 37°C for 30 min. Next, cell lysates were centrifuged at 18,000 rcf at room temperature (RT) for 30 min. The clear supernatant was used for determining protein concentration with BCA assay as per manufacturer’s instructions (thermo scientific). Lysates corresponding to 70 µg (NMCM) or 100 µg (RPTEC) of protein of each sample were subjected to carbamidomethylation i.e. reduction of disulfide bonds with 10 mM DTT and incubation at 56°C for 30 min followed by alkylation of free thiol groups with 30 mM IAA and incubation at RT for 30 min in the dark. Sample cleaning and proteolysis (trypsin) were performed using the S-Trap mini protocol as previously described (Hentschel et al. 2021). Next, the digests were completely dried in a SpeedVac and the dried peptides were resolubilized in 0.1% TFA followed by evaluation of digestion efficiency on a Monolithic HPLC (Burkhart et al. 2012). Lastly, the peptide concentration of each sample was determined using a Nanodrop spectrophotometer (Thermo Scientific).

***LC-MS/MS analysis***

Peptides corresponding to ~0.75 µg of all 33 samples i.e. 5 replicates per condition of NMCM and 6 replicates per condition of RPTEC, respectively were individually analyzed by nano-LC-MS/MS using an Ultimate 3000 nano RSLC system coupled to a Q Exactive HF mass spectrometer (both Thermo Scientific). Peptides were preconcentrated on a 100 µm x 2 cm C18 trapping column for 5 min using 0.1% TFA (v/v) with a flow rate of 20 µL/min followed by separation on a 75 µm x 50 cm C18 main column (both Acclaim Pepmap nanoviper, Thermo Scientific) with a 120 min LC gradient ranging from 3-35% of B (84% ACN in 0.1% FA) at a flow rate of 250 nL/min. The Q Exactive HF was operated in data-dependent acquisition mode and MS survey scans were acquired from m/z 300 to 1500 at a resolution of 60000 using the polysiloxane ion at m/z 371.1012 as lock mass (Olsen et al. 2005). The fifteen most intense ions were isolated with a 1.2 m/z window and fragmented by higher-energy collisional dissociation with a normalized collision energy of 27%, taking into account a dynamic exclusion of 20 s. MS/MS scans were acquired at a resolution of 15000. Automatic gain control target values and fill times were set to 3 × 10^6^ and 120 ms for MS and 5 × 10^4^ and 200 ms for MS/MS, respectively, with a minimum intensity threshold of 7.5 × 10^4^ considering an under fill ratio of 30%.

***Label-free quantitative data analysis***

Data analysis of each sample set i.e. NMCM and RPTEC was performed with the Proteome Discoverer (PD) software 2.3 using the precursor-based label-free quantitation workflow nodes. MS/MS spectra of the respective species data set were searched against their corresponding Uniprot databases i.e. mouse with 17019 target entries (downloaded 08.10.2019) and human with target entries 20364 (downloaded on 21.11.2019) using Mascot 2.6.1 (Matrix Science). Trypsin with a maximum of two missed cleavages was selected as enzyme. Carbamidomethylation of Cys was set as fixed and oxidation of Met was selected as variable modification. MS and MS/MS tolerances were set to 10 ppm and 0.02 Da, respectively. False discovery rate (FDR) validation on the peptide-spectrum match (PSM) level was done using Percolator node. Peak and feature detection were done by the "Minora" feature detector node using default parameters. In the Consensus workflow of PD, the peptide and protein filters were set to a FDR of 1% and default settings of "Feature Mapper" node were employed. For the "Precursor Ions Quantifier" node, only *unique* peptides were set to use and "Precursor Abundance Based On" was set to *intensity*. "Normalization Mode" was set to *total peptide amount* and for "Scaling" the normalized abundances; *on all average* was selected. "Protein Abundance Calculation" was done by using the *summed abundances* and "Protein Ratio Calculation" was set to *protein abundance based*. For missing values, the "Imputation Mode" was set to *low abundance resampling*. Statistical test was based on ANOVA t-test (Background Based) and the *p*-values were adjusted using the Benjamini-Hochberg method. In both data sets, only those proteins that were quantified with (i) high confidence settings i.e. 1% FDR on protein, peptide and PSM level (ii) ≥ 2 unique peptides were considered for further evaluation.

*Hypertrophy assessment*

Neonatal mouse cardiomyocytes (NMCM) were isolated from newborn mice and seeded on glass coverslips (coated with poly-lysine). Cells were treated with angiotensin II (200 nM; Sigma) or insulin-like growth factor (300 pM; Sigma) for 24 h with co-incubation with remdesivir (6,25 µM) as indicated. Cardiomyocytes were fixed with ice-cold methanol (10 min, -20 °C) and permeabilized with Triton-X (0,2% (v/v) in DPBS; 10 min at room temperature). NMCM were incubated with primary antibody directed against sarcomere protein α-actinin (Sigma, A7811; 1:1.000). For detection Cy-3 conjugated secondary antibody (Jackson ImmunoResearch, 715-165-150; 1:250) was used. Cell nuclei were stained with DAPI (4′,6-diamidino-2-phenylindole). For analysis of samples, a Leica TCS SP5 confocal microscope (Leica Microsystems) was used. Excitation of Cy-3 was performed at 488 nm, emission was measured between 570-650 nm. Excitation of DAPI was performed at 405 nm, emission was detected between 430-485 nm.

Cell sizes were analyzed using ImageJ software (Schneider et al. 2012) by manually circling of cardiomyocytes. At least 60 cells per condition and per experiment.

*Determination of mitochondrial DNA copy number*

Genomic DNA was isolated from 0.5 * 10^6^ NMCM, RPTEC/TERT1, NRK-52E or H9c2 per well using the DNeasy Blood&Tisssue Kit (Qiagen) according to manufacturer’s instructions. 25 ng DNA was amplified in a 384 well format by the C1000 Touch Thermal Cycler using the CFX384™ Real-Time System (Biorad) using the program 3 min 95 °C, 45x(10 sec. 95 °C, 10 sec. 60 °C, 20 sec. 65 °C), 10 sec. 65 °C. Primers are listed in Table 1 and used at a concentration of 1 µM in diluted Eva Green Mix (Biorad) according to manufacturer’s instructions. Amplification efficiency was determined at every run using 5 1:1 dilutions of pooled DNA. Only runs with efficiencies over 100 % were included. Samples were run in triplicates. Only CT-values from triplicates with SD < 0.3 were considered. The mitochondrial copy number was calculated using the formula:

*
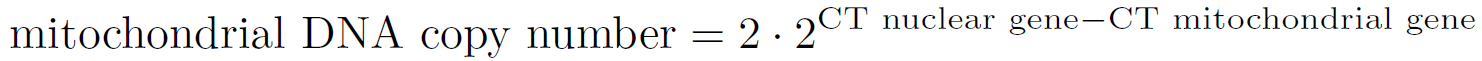
*

according to Quiros et al. 2017(Quiros et al. 2017).

| *Mouse (Quiros et al. 2017)* | *16S rRNA (mitochondrial)* | 5’-CCGCAAGGGAAAGATGAAAGAC-3’ |
| --- | --- | --- |
|  |  | 5’-TCGTTTGGTTTCGGGGTTTC-3’ |
|  | *ND1 (mitochondrial)* | 5’-CTAGCAGAAACAAACCGGGC-3’ |
|  |  | 5’-CCGGCTGCGTATTCTACGTT-3’ |
|  | *HK2 (nuclear)* | 5’-GCCAGCCTCTCCTGATTTTAGTGT-3’ |
|  |  | 5’-GGGAACACAAAAGACCTCTTCTGG-3’ |
| *rat* | *COII (mitochondrial)(Piantadosi and Suliman 2006)* | 5′-TGAGCCATCCCTTCACTAGG-3′ |
|  |  | 5′-TGAGCCGCAAATTTCAGAG-3′ |
|  | *ND1 (mitochondrial)(Ikeda et al. 2015)* | 5'-ACTCCCTATTCGGAGCCCTA-3' |
|  |  | 5'-GGAGCTCGATTTGTTTCTGC-3' |
|  | *ATIII (nuclear)(Ikeda et al. 2015)* | 5'-GCCCTGAGAACACAAGGAAG-3' |
|  |  | 5'-CTCTCCCACACGCCTGTATT-3' |
| *human* | *mitochondrial DNA* | 5’-CACCCAAGAACAGGGTTTGT-3’ |
|  |  | 5’-TGGCCATGGGTATGTTGTTA-3’ |
|  | *B2M (nuclear)* | 5’-TCTCTGCTCCCCACCTCTAAGT-3’ |
|  |  | 5’-TGCTGTCTCCATGTTTGATGTATCT-3’ |

**Table S1**. Primer sequences used for the assessment of mitochondrial copy number

*Confluence analysis of zenCell Owl pictures*

15000 H9c2 or 10000 NRK-52E cells were seeded in standard culture medium in 24-well plates. 1-2 hours after seeding cells were treated with remdesivir and placed on the zenCELL owl microscope (innoME, Espelkamp) in an incubator with 37 °C, 5 % CO2. Pictures were taken from each well every 2 hours for several days and processed for confluence analysis by applying following macro in Image J Fiji RRID: SRC_002285 (Schindelin et al. 2012):

run("Enhance Contrast...", "saturated=0 equalize");

run("Canny Edge Detector", "gaussian=1.25 low=0.1 high=8");

run("Maximum...", "radius=4");

run("Options...", "iterations=10 count=3 pad do=Close");

run("Options...", "iterations=25 count=3 do=Open");

run("Options...", "iterations=2 count=3 pad do=Erode");

run("Measure");

The percentage of covered area was plotted against time in the GraphPad Prism 9.1.2 (GraphPad Software) and the slope of the linear regression was used as parameter for proliferation rate. Videos were generated from the pictures by the zenCELL owl software (innoME).

**Supplementary Tables**

**Table S2**. Proteins in NMCM > 2-fold differentially regulated by remdesivir

Proteins > 2-fold up- or down-regulated by 12 h treatment with 9 µM remdesvir (adj. P-value < 0.05) were selected out of 2875 relatively quantified proteins. Proteins also significantly regulated by 0.2 µM antimycin A are highlighted in grey.

**Table S3**. Proteins in RPTEC/TERT1 > 2-fold differentially regulated by remdesivir

Proteins > 2-fold up- or down-regulated by 12 h treatment with 9 µM remdesvir (adj. P-value < 0.05) were selected out of 3801 relatively quantified proteins. Proteins also significantly regulated by 0.2 µM antimycin A are highlighted in grey.

**Supplementary figure legends**

**Fig. S1** The effect of remdesivir on growth and viability of H9c2 and NRK-52E cells

(**a**) H9c2 cells cells were treated with increasing concentrations of remdesivir 2 h after seeding. The proliferation-slope of H9c2 cells was derived from increasing well coverage values over 86 h. Data were normalized to the mean of solvent controls (dmso = 100 %, dashed line) (n = 4-6). RM one-way ANOVA, Dunnett‘s multiple comparisons test. (**b**) H9c2 (n = 2), (**c**) NRK-52E (n = 7-8) were treated with increasing concentrations of remdesivir for 24 h and LDH-activity was measured in the supernatants (LDH-activity after 10-20 % dmso-treatment was defined as 100 % cytotoxicity). (**d**) FACS gating strategy in NRK-52E cells for single dead cells and cells in G1 or G2/M phase. (**e**) Quantification of the frequency of dead cells in NRK-52E cells treated with remdesivir for 24 h measured by FACS (n = 6; Kruskal-Wallis-test with Dunn‘s multiple comparisons test) * p < 0.05, ** p < 0.01, *** p< 0.001

**Fig. S2** Beating behaviour of NMCM changed by late treatment with remdesivir

NMCM were treated with 3.1 or 6.25 µM of remdesvir or with solvent control (dmso). The parameters beat rate (**a**), pulse width 90 % (**b**), beat amplitude (**c**) and base impedance (**d**) were calculated from 20 sec. impedance recordings every hour after treatment. Data were normalized to solvent control (dmso = 100 %) in each experiment, n = 4-7 independent experiments run with 4-6 technical replicates (wells) each. Two-way ANOVA, Dunnett‘s multiple comparisons test * p < 0.05, ** p < 0.01, ** p < 0.001

**Fig. S3** The effect of remdesivir on different parameters of mitochondrial function

(**a**) H9c2, (**b**) NRK-52E or (**c**) RPTEC/TERT1 cells were treated with increasing concentrations of remdesivir for 24 h. 1 h after remdesivir removal the oxygen consumption rate (OCR) was measured during injections of the inhibitors of the respiratory chain oligomycin, FCCP or rotenone/antimycin A. Different parameters were calculated from the OCR as described in material and methods (n = 5-6). Data were normalized to the solvent controls (dmso = 100 %; dashed line) Kruskal-Wallis-test with Dunn‘s multiple comparisons test * p < 0-05, ** p < 0.01, *** p < 0.001

**Fig. S4** Reversibility of remdesivir’s effect on mitochondrial function

(**a**) H9c2, (**b**) NRK-52E or (**c**) RPTEC/TERT1 cells were treated with 12 µM (**a, c**) or 3 µM (**b**) of remdesivir for 24 h. 1 h after remdesivir removal the oxygen consumption rate (OCR) was measured every 15 min for 12 h. The OCR, which was normalized only to the cell number (left) and the OCR-ratio of remdesivir/dmso (right) over time is shown (mean, SD, n = 6).

**Fig. S5** Effect of remdesivir on mitochondrial DNA copy number

(**a**) NMCM were treated with increasing concentrations of remdesivir for 24 h (n = 2-3) or 7 days (n = 3). DNA was isolated and qPCR for mouse mitochondrial genes *Nd1* and *16S rRNA* and nuclear gene *Hk2* was performed. (**b**) H9c2 (n = 2-3), (**c**) NRK-52E (n = 2) or (**d**, **e**) RPTEC/TERT1 cells (were treated with remdesivir (**b**, **c, d**) or ddC (**e**; n = 3) repetetively every 3rd day for 14 days. DNA was isolated and qPCR for rat mitochondrial genes *Nd1* and *CoII* and nuclear gene *AtIII* (**b**, **c**) or for human mitochondrial DNA and the nuclear gene *B2M* (**d**, **e**) was performed. (**a**-**e**) The mitochondrial copy number was normalized to the solvent control (dmso = 100 %, dashed line) Kruskal-Wallis-test with Dunn‘s multiple comparisons test * p < 0.05, ** p < 0.01, *** p < 0.001

**Fig. S6** The effect of different metabolic condtions on the toxicity of remdesivir and antimycin A

Cells were treated with increasing concentrations of remdesivir (**a - c**) or antimycin A (**d**) and viability was determined by ATP-measurement after indicated time periods. (**a**) H9c2 cells were cultured and treated in medium containing 25 mM glucose, 5 mM glucose or 10 mM galactose (n = 5). (**b**) RPTEC/TERT1 cells were cultured and treated in medium containing 10 mM glucose or 10 mM galactose. Within the 120h period cells were retreated once (n = 5-7). (**c**) NRK-52E cells were cultured and treated in medium containing 25 mM glucose or 10 mM galactose (n = 7). (**d**) Indicated cell lines were treated with increasing concentrations of antimycin A for 72 h (H9c2: n = 3, RPTEC: n = 7, NRK-52E: n = 6) Data were normalized to the solvent controls (dmso = 100 %, dashed line). Kruskal-Wallis-test with Dunn‘s multiple comparisons test * p < 0.05, ** p < 0.01, *** p < 0.001

**Fig. S7** Effect of remdesivir on the hypertrophic response of NMCM

NMCM were treated with 200 nM angiotensin II (AngII) or 300 pM insulin-like growth factor (IGF) with or without co-treatment with 6.25 µM remdesivir for 24 h. Cell sizes were determined by microscopical examination and normalized to the solvent control (dmso = 1). Kruskal-Wallis-test with Dunn‘s test for multiple comparisons (n = 6; * p < 0.05)

**Fig. S8** Comparison of the relative sensitivity towards mitotoxic agents between different cell lines

(**a**) NRK-52E (n = 9), RPTEC/TERT1 (n = 7) or H9c2 cells (n = 3) were treated with increasing doses of antimycin A or solvent control (dmso) for 24 h and lactate was quantified in the supernatants. (**b**) NRK-52E cells were treated with remdesivir, antimycin A (AA) or solvent control (dmso) for 24 h and ROS-release was quantified in the supernatants by fluorescent detection of H_2_O_2_ (n = 6). (**a**, **b**) RM-ANOVA with Dunnet‘s multiple comparisons test * p < 0.05, *** p < 0.001 (**c**) indicated cell types were treated with 0.1 % dmso for 24 h. Lactate concentrations were determined in supernatants. *Data are also shown in a normalized way in Fig. 3 a (H9c2) and Fig. 3 c, e (NMCM)*. One-way ANOVA * p < 0.05

**Supplementary Videos**

H9c2 or NRK-52E cells were seeded in 24-well plate at low density, treated with 12.5 µM remdesivir or solvent control (dmso) and monitored by light microscopy every two hours.

**Supplementary References**

Bouitbir J, Panajatovic MV, Krähenbühl S (2022) Mitochondrial Toxicity Associated with Imatinib and Sorafenib in Isolated Rat Heart Fibers and the Cardiomyoblast H9c2 Cell Line. Int J Mol Sci 23(4) doi:10.3390/ijms23042282

Burkhart JM, Schumbrutzki C, Wortelkamp S, Sickmann A, Zahedi RP (2012) Systematic and quantitative comparison of digest efficiency and specificity reveals the impact of trypsin quality on MS-based proteomics. Journal of Proteomics 75(4):1454-1462 doi:10.1016/j.jprot.2011.11.016

de Larco JE, Todaro GJ (1978) Epithelioid and fibroblastic rat kidney cell clones: epidermal growth factor (EGF) receptors and the effect of mouse sarcoma virus transformation. Journal of cellular physiology 94(3):335-42 doi:10.1002/jcp.1040940311

Han JW, Kang C, Kim Y, Lee MG, Kim JY (2020) Isoproterenol-induced hypertrophy of neonatal cardiac myocytes and H9c2 cell is dependent on TRPC3-regulated Ca(V)1.2 expression. Cell calcium 92:102305 doi:10.1016/j.ceca.2020.102305

Hentschel A, Czech A, Münchberg U, et al. (2021) Protein signature of human skin fibroblasts allows the study of the molecular etiology of rare neurological diseases. Orphanet J Rare Dis 16(1):73-73 doi:10.1186/s13023-020-01669-1

Huu D-N, Rosenblum EN, Zeigel RF (1966) Persistent infection of a rat kidney cell line with Rauscher murine leukemia virus. Journal of bacteriology 92(4):1133-40 doi:10.1128/jb.92.4.1133-1140.1966

Ikeda M, Ide T, Fujino T, et al. (2015) Overexpression of TFAM or twinkle increases mtDNA copy number and facilitates cardioprotection associated with limited mitochondrial oxidative stress. PLoS One 10(3):e0119687 doi:10.1371/journal.pone.0119687

Janssen PM, Biesiadecki BJ, Ziolo MT, Davis JP (2016) The Need for Speed: Mice, Men, and Myocardial Kinetic Reserve. Circ Res 119(3):418-21 doi:10.1161/circresaha.116.309126

Kaja S, Payne AJ, Singh T, Ghuman JK, Sieck EG, Koulen P (2015) An optimized lactate dehydrogenase release assay for screening of drug candidates in neuroscience. J Pharmacol Toxicol Methods 73:1-6 doi:10.1016/j.vascn.2015.02.001

Kimes BW, Brandt BL (1976) Properties of a clonal muscle cell line from rat heart. Experimental cell research 98(2):367-81 doi:10.1016/0014-4827(76)90447-x

Kuznetsov AV, Javadov S, Sickinger S, Frotschnig S, Grimm M (2015) H9c2 and HL-1 cells demonstrate distinct features of energy metabolism, mitochondrial function and sensitivity to hypoxia-reoxygenation. Biochim Biophys Acta 1853(2):276-84 doi:10.1016/j.bbamcr.2014.11.015

Lalowski MM, Bjork S, Finckenberg P, et al. (2018) Characterizing the Key Metabolic Pathways of the Neonatal Mouse Heart Using a Quantitative Combinatorial Omics Approach. Front Physiol 9:365 doi:10.3389/fphys.2018.00365

Lash LH, Putt DA, Hueni SE, et al. (2002) Cellular energetics and glutathione status in NRK-52E cells: toxicological implications. Biochem Pharmacol 64(10):1533-46 doi:10.1016/s0006-2952(02)01360-6

Lechner CA (2014) Inaugural-Dissertation, Nierenzellen als In-vitro-Modell zur Evaluierung der renalen Sekretion von Arzneistoffkandidaten. Ruprecht-Karls-Universität

Li S, Jiang J, Fang J, et al. (2021) Naringin protects H9C2 cardiomyocytes from chemical hypoxia‑induced injury by promoting the autophagic flux via the activation of the HIF‑1α/BNIP3 signaling pathway. Int J Mol Med 47(6) doi:10.3892/ijmm.2021.4935

Lin Z, Will Y (2012) Evaluation of drugs with specific organ toxicities in organ-specific cell lines. Toxicol Sci 126(1):114-27 doi:10.1093/toxsci/kfr339

Liu Y, Nguyen P, Baris TZ, Poirier MC (2012) Molecular analysis of mitochondrial compromise in rodent cardiomyocytes exposed long term to nucleoside reverse transcriptase inhibitors (NRTIs). Cardiovasc Toxicol 12(2):123-34 doi:10.1007/s12012-011-9148-5

Liu Y, Shim E, Crespo-Mejias Y, et al. (2015) Cardiomyocytes are Protected from Antiretroviral Nucleoside Analog-Induced Mitochondrial Toxicity by Overexpression of PGC-1α. Cardiovasc Toxicol 15(3):224-31 doi:10.1007/s12012-014-9288-5

Liu Y, Shim E, Nguyen P, Gibbons AT, Mitchell JB, Poirier MC (2014) Tempol protects cardiomyocytes from nucleoside reverse transcriptase inhibitor-induced mitochondrial toxicity. Toxicol Sci 139(1):133-41 doi:10.1093/toxsci/kfu034

Lund KC, Peterson LL, Wallace KB (2007) Absence of a universal mechanism of mitochondrial toxicity by nucleoside analogs. Antimicrob Agents Chemother 51(7):2531-9 doi:10.1128/AAC.00039-07

Lund KC, Wallace KB (2004) Direct effects of nucleoside reverse transcriptase inhibitors on rat cardiac mitochondrial bioenergetics. Mitochondrion 4(2-3):193-202 doi:10.1016/j.mito.2004.06.009

Lynx MD, Kang BK, McKee EE (2008) Effect of AZT on thymidine phosphorylation in cultured H9c2, U-937, and Raji cell lines. Biochem Pharmacol 75(8):1610-5 doi:10.1016/j.bcp.2008.01.006

Miyoshi T, Nakamura K, Amioka N, et al. (2022) LCZ696 ameliorates doxorubicin-induced cardiomyocyte toxicity in rats. Sci Rep 12(1):4930 doi:10.1038/s41598-022-09094-z

Olsen JV, de Godoy LMF, Li G, et al. (2005) Parts per Million Mass Accuracy on an Orbitrap Mass Spectrometer via Lock Mass Injection into a C-trap. Molecular & Cellular Proteomics 4(12):2010-2021

Piantadosi CA, Suliman HB (2006) Mitochondrial transcription factor A induction by redox activation of nuclear respiratory factor 1. J Biol Chem 281(1):324-33 doi:10.1074/jbc.M508805200

Quiros PM, Goyal A, Jha P, Auwerx J (2017) Analysis of mtDNA/nDNA Ratio in Mice. Curr Protoc Mouse Biol 7(1):47-54 doi:10.1002/cpmo.21

Schindelin J, Arganda-Carreras I, Frise E, et al. (2012) Fiji: an open-source platform for biological-image analysis. Nature methods 9(7):676-82 doi:10.1038/nmeth.2019

Schneider CA, Rasband WS, Eliceiri KW (2012) NIH Image to ImageJ: 25 years of image analysis. Nature Methods. 9(7):671-675 doi:doi:10.1038/nmeth.2089

Simon BR, Wilson MJ, Wickliffe JK (2014) The RPTEC/TERT1 cell line models key renal cell responses to the environmental toxicants, benzo[a]pyrene and cadmium. Toxicology reports 1:231-242 doi:10.1016/j.toxrep.2014.05.010

Wang L, Liu S, Gao C, et al. (2022) Arsenic trioxide-induced cardiotoxicity triggers ferroptosis in cardiomyoblast cells. Human & experimental toxicology 41:9603271211064537 doi:10.1177/09603271211064537

Watkins SJ, Borthwick GM, Arthur HM (2011) The H9C2 cell line and primary neonatal cardiomyocyte cells show similar hypertrophic responses in vitro. In vitro cellular & developmental biology Animal 47(2):125-31 doi:10.1007/s11626-010-9368-1

Webster DR, Patrick DL (2000) Beating rate of isolated neonatal cardiomyocytes is regulated by the stable microtubule subset. Am J Physiol Heart Circ Physiol 278(5):H1653-61 doi:10.1152/ajpheart.2000.278.5.H1653

Wieser M, Stadler G, Jennings P, et al. (2008) hTERT alone immortalizes epithelial cells of renal proximal tubules without changing their functional characteristics. American journal of physiology Renal physiology 295(5):F1365-75 doi:10.1152/ajprenal.90405.2008
