# Supplementary material for: The potential of remdesivir to affect function, metabolism and proliferation of cardiac and kidney cells in vitro
Source: Arch Toxicol. 2022 May 17;96(8):2341–60. doi: 10.1007/s00204-022-03306-1 (PMC9110936; doi:10.1007/s00204-022-03306-1)
Supplement: Supplementary file 2 — Supplementary file2 (PDF 929 kb) [file 204_2022_3306_MOESM2_ESM.pdf]

Figure S1

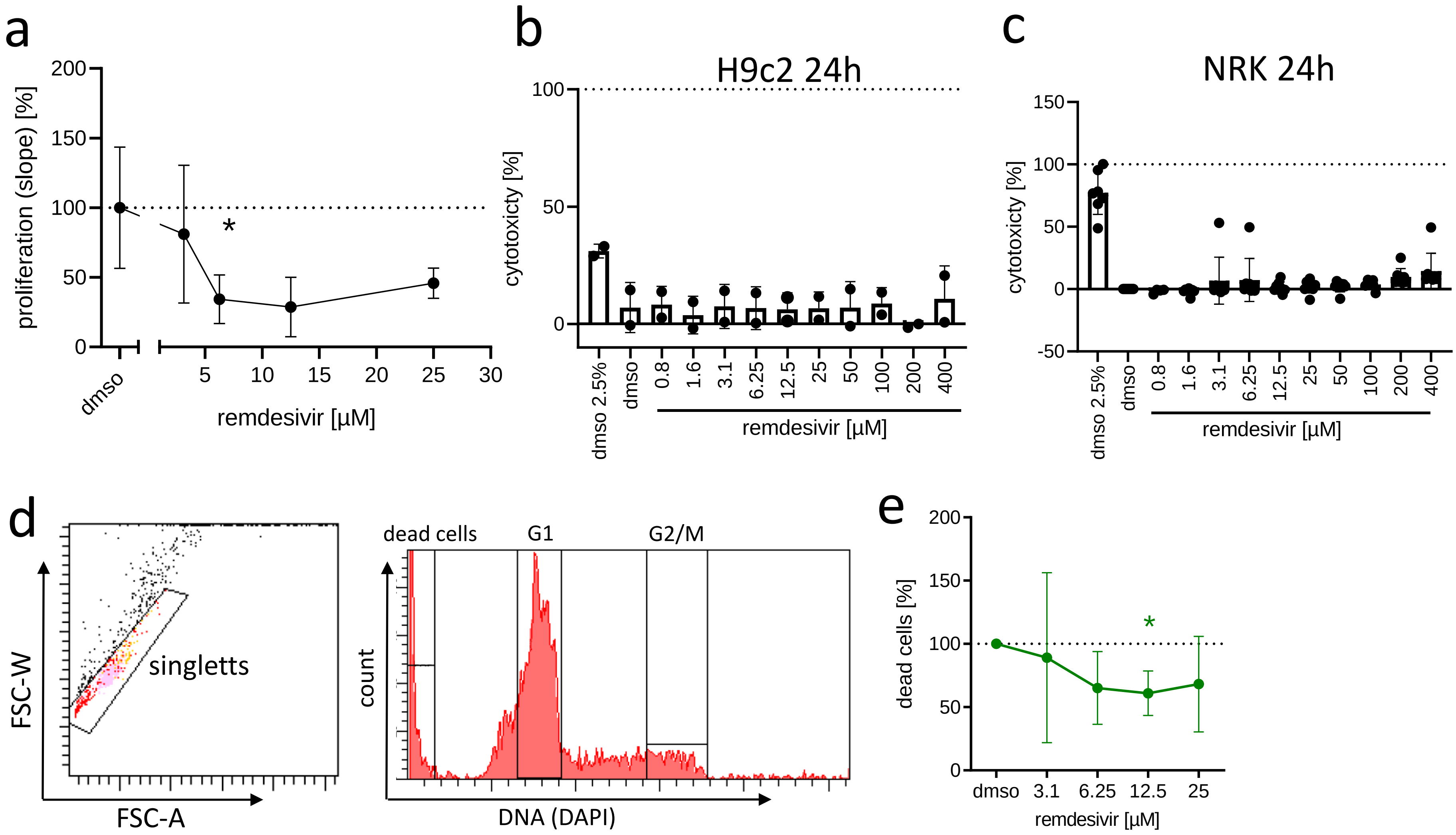

Fig. S1 The effect of remdesivir on growth and viability of H9c2 and NRK-52E cells
(**a**) H9c2 cells cells were treated with increasing concentrations of remdesivir 2 h after seeding. The proliferation-slope of H9c2 cells was derived from increasing well coverage values over 86 h. Data were normalized to the mean of solvent controls (dmso = 100 %, dashed line). N = 4-6. RM one-way ANOVA, Dunnett‘s multiple comparisons test. (**b**) H9c2 (n = 2) or (**c**) NRK-52E (n = 7-8) were treated with increasing concentrations of remdesivir for 24 h and LDH-activity was measured in the supernatants (LDH-activity after 10-20 % dmso-treatment was defined as 100 % cytotoxicity). (**d**) FACS gating strategy in NRK-52E cells for single dead cells and cells in G1 or G2/M phase. (**e**) Quantification of the frequency of dead cells in NRK-52E cells treated with remdesivir for 24 h measured by FACS (n = 6; Kruskal-Wallis-test with Dunn‘s multiple comparisons test) * $p < 0.05$, ** $p < 0.01$, *** $p < 0.001$

Figure S2

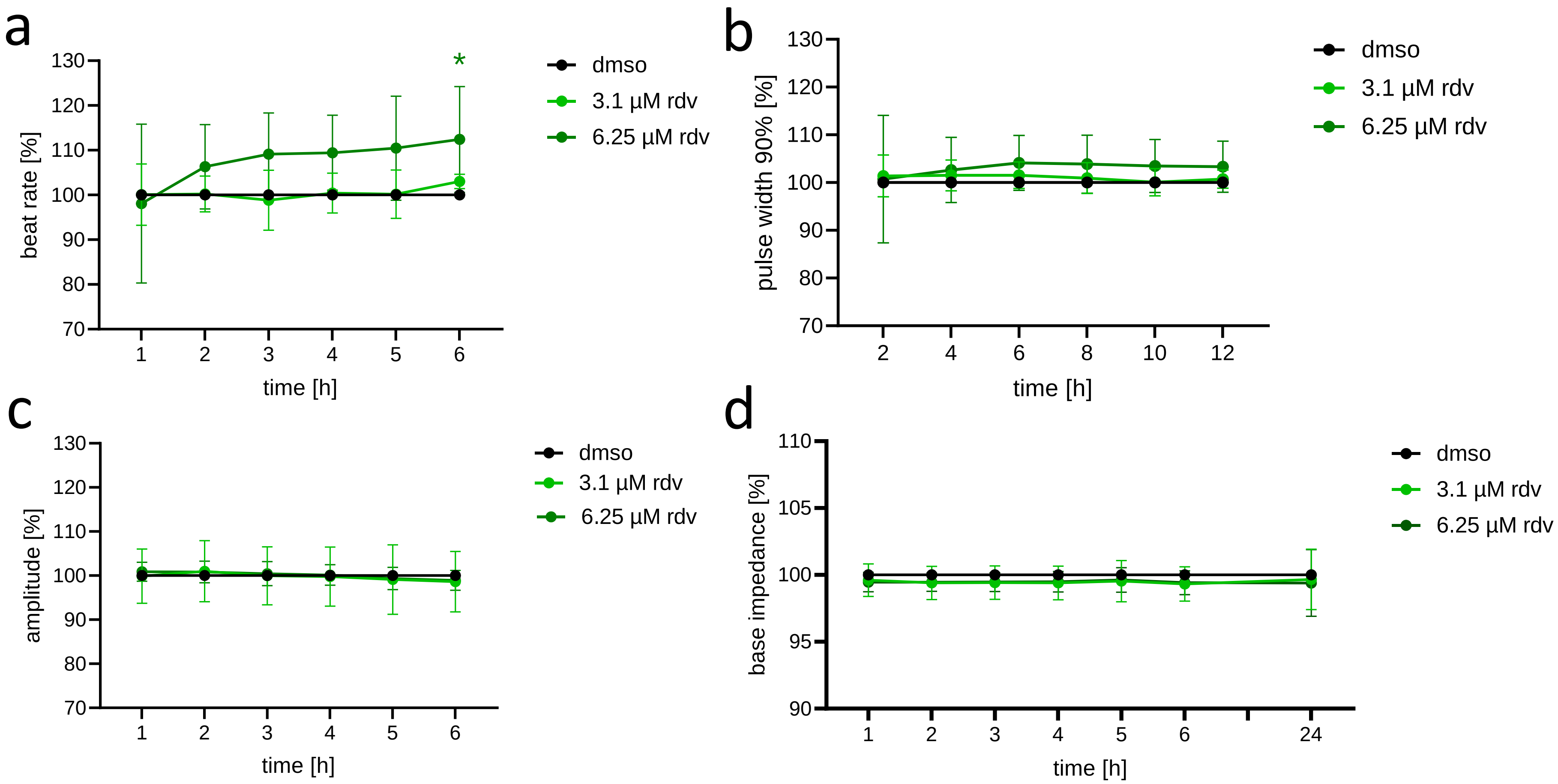

Fig. S2 Beating behaviour of NMCM changed by late treatment with remdesivir
NMCM were treated with 3.1 or 6.25 µM of remdesvir or with solvent control (dmso). The parameters beat rate (**a**), pulse width 90 % (**b**), beat amplitude (**c**) and base impedance (**d**) were calculated from 20 sec. impedance recordings every hour after treatment. Data were normalized to solvent control (dmso = 100 %) in each experiment, n = 4-7 independent experiments run with 4-6 technical replicates (wells) each. Two-way ANOVA, Dunnett's multiple comparisons test * $p < 0.05$, ** $p < 0.01$, ** $p < 0.001$

Figure S3

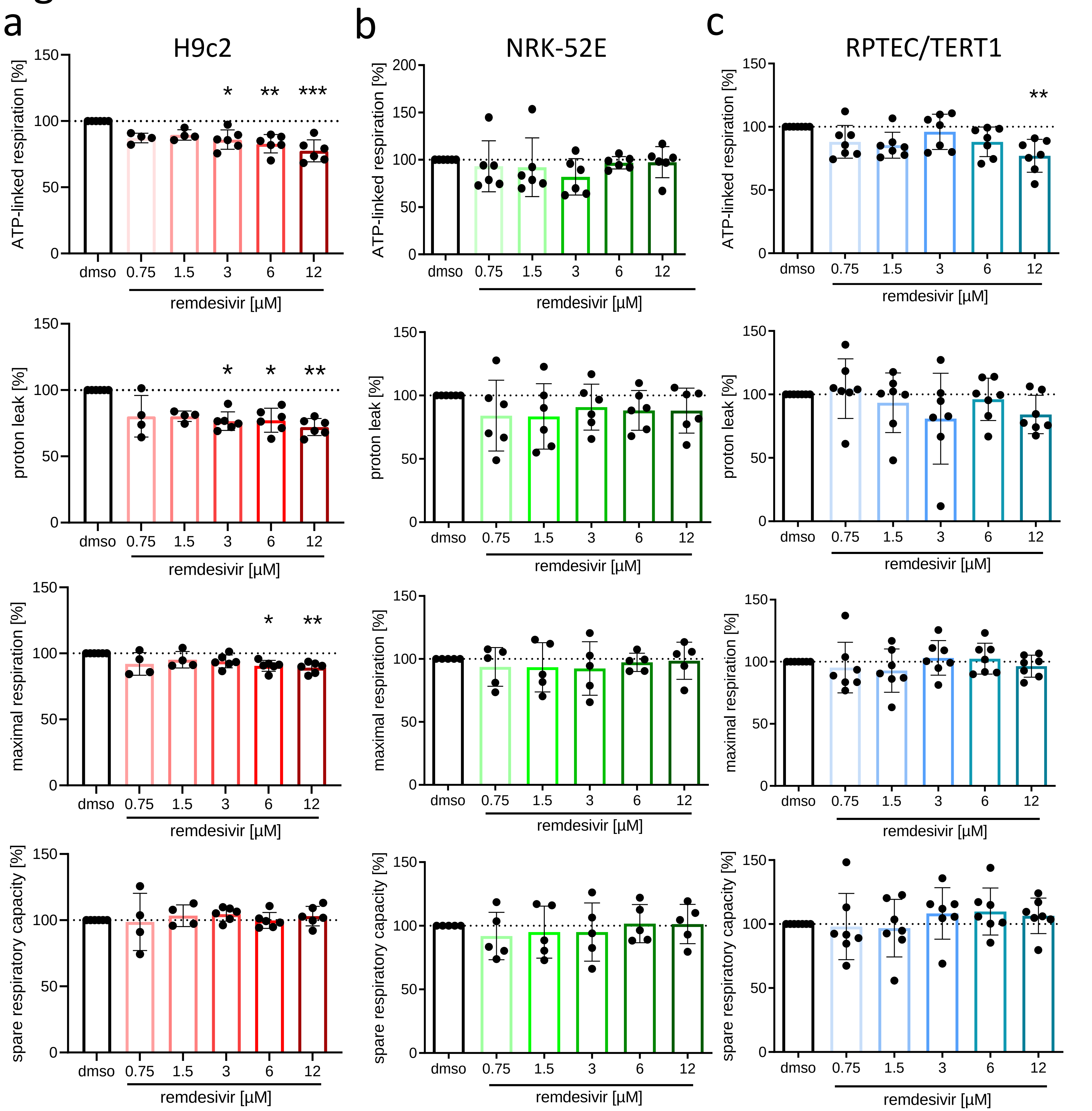

Fig. S3 The effect of remdesivir on different parameters of mitochondrial function
(**a**) H9c2, (**b**) NRK-52E or (**c**) RPTEC/TERT1 cells were treated with increasing concentrations of remdesivir for 24 h. 1 h after remdesivir removal the oxygen consumption rate (OCR) was measured during injections of the inhibitors of the respiratory chain oligomycin, FCCP or rotenone/antimycin A. Different parameters were calculated from the OCR as described in material and methods (n = 5-6). Data were normalized to the solvent controls (dmso = 100 %; dashed line) Kruskal-Wallis-test with Dunn‘s multiple comparisons test * p < 0-05, ** p < 0.01, *** p < 0.001

Figure S4

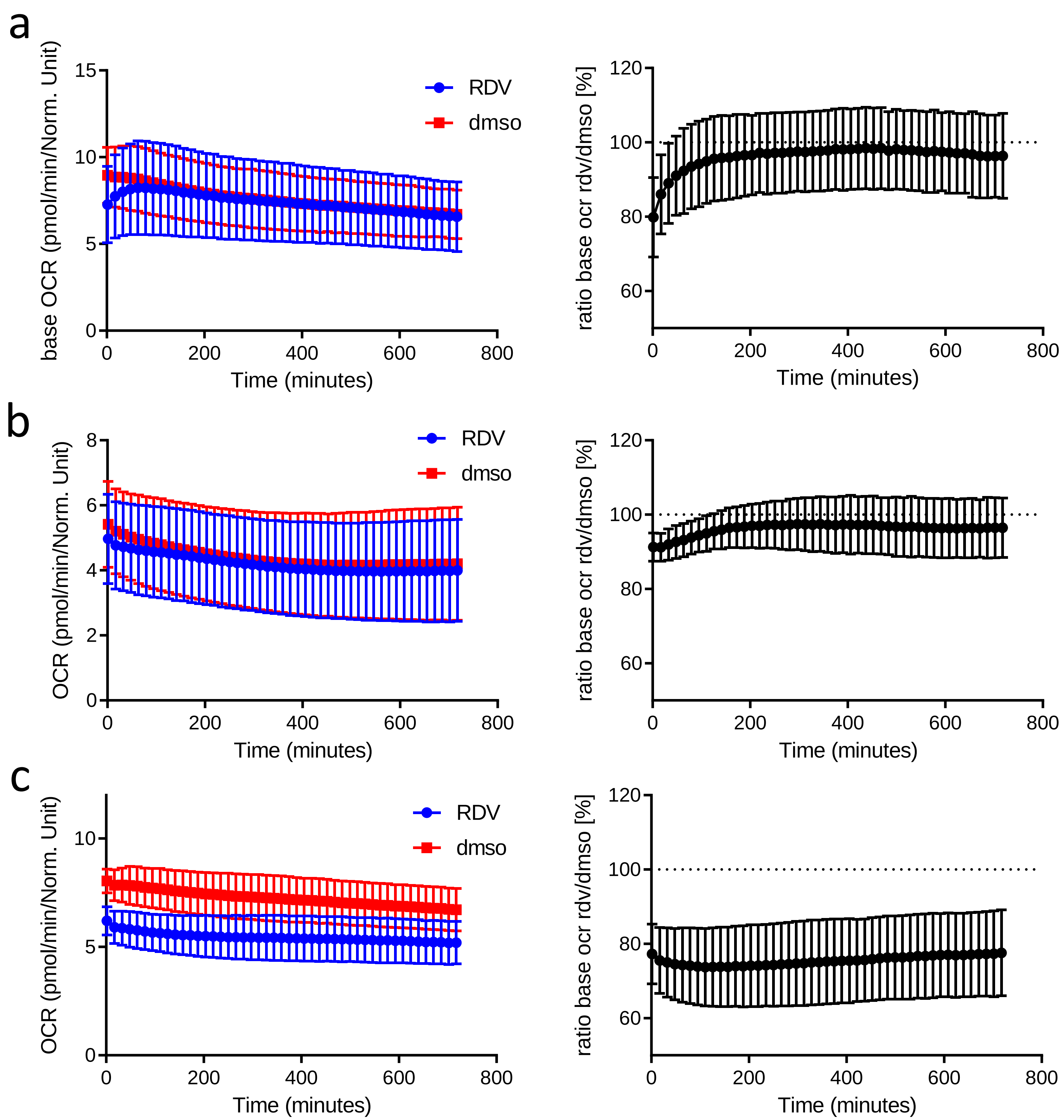

Fig. S4 Reversibility of remdesivir`s effect on mitochondrial function
(**a**) H9c2, (**b**) NRK-52E or (**c**) RPTEC/TERT1 cells were treated with 12 µM (**a, c**) or 3 µM (**b**) of remdesivir for 24 h. 1 h after remdesivir removal the oxygen consumption rate (OCR) was measured every 15 min for 12 h. The OCR, which was normalized only to the cell number (left) and the OCR-ratio of remdesivir/dmso (right) over time is shown (mean, SD, n = 6).

Figure S5

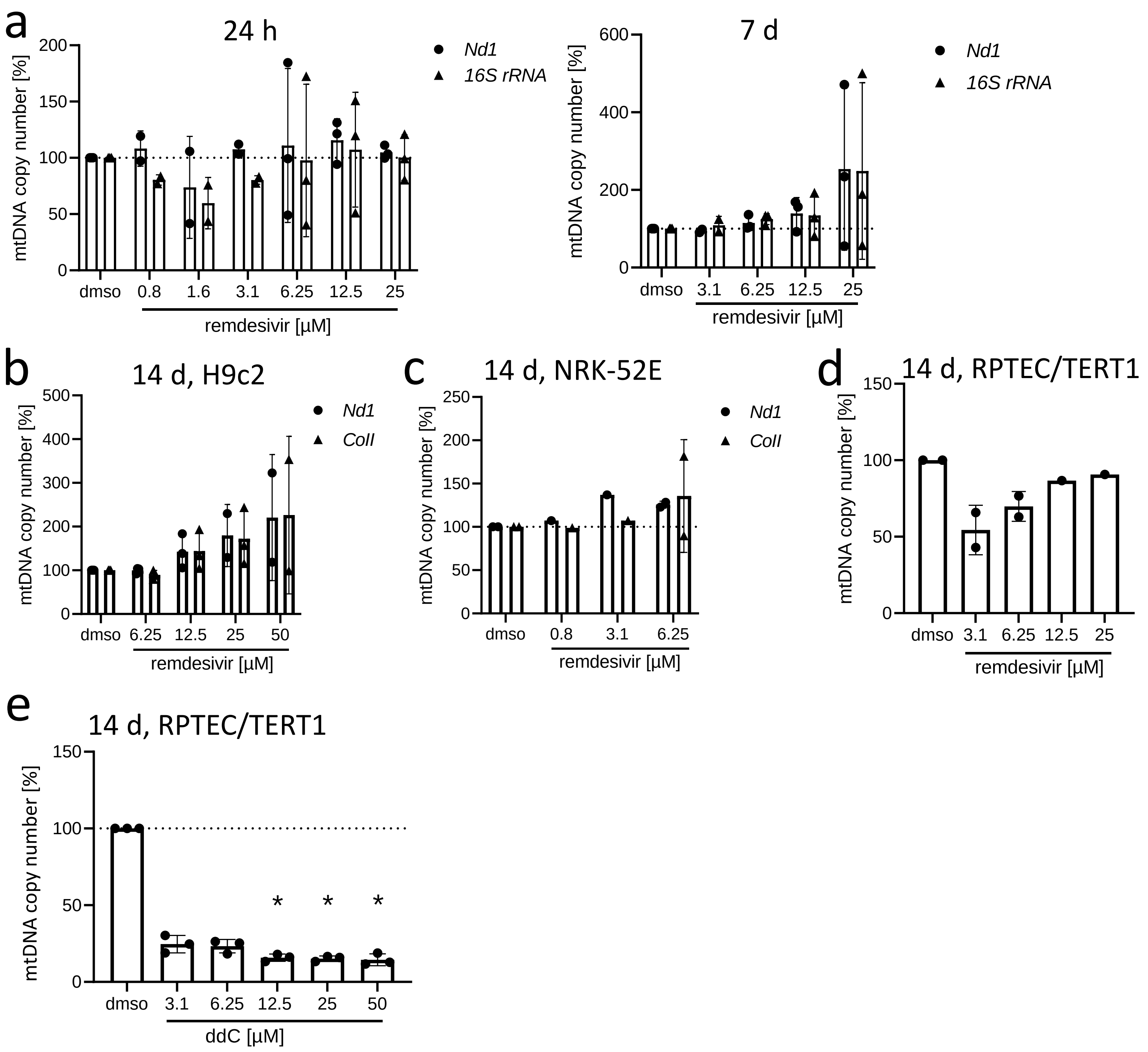

Fig. S5 Effect of remdesivir on mitochondrial DNA copy number
(**a**) NMCM were treated with increasing concentrations of remdesivir for 24 h (n = 2-3) or 7 days (n = 3). DNA was isolated and qPCR for mouse mitochondrial genes *Nd1* and *16S rRNA* and nuclear gene *Hk2* was performed. (**b**) H9c2 (n = 2-3), (**c**) NRK-52E (n = 2) or (**d**, **e**) RPTEC/TERT1 cells (were treated with remdesivir (**b**, **c, d**) or ddC (**e**; n = 3) repetetively every 3rd day for 14 days. DNA was isolated and qPCR for rat mitochondrial genes *Nd1* and *CoII* and nuclear gene *AtIII* (**b**, **c**) or for human mitochondrial DNA and the nuclear gene *B2M* (**d**, **e**) was performed. (**a**-**e**) The mitochondrial copy number was normalized to the solvent control (dmso = 100 %, dashed line) Kruskal-Wallis-test with Dunn's multiple comparisons test * $p < 0.05$, ** $p < 0.01$, *** $p < 0.001$

Figure S6

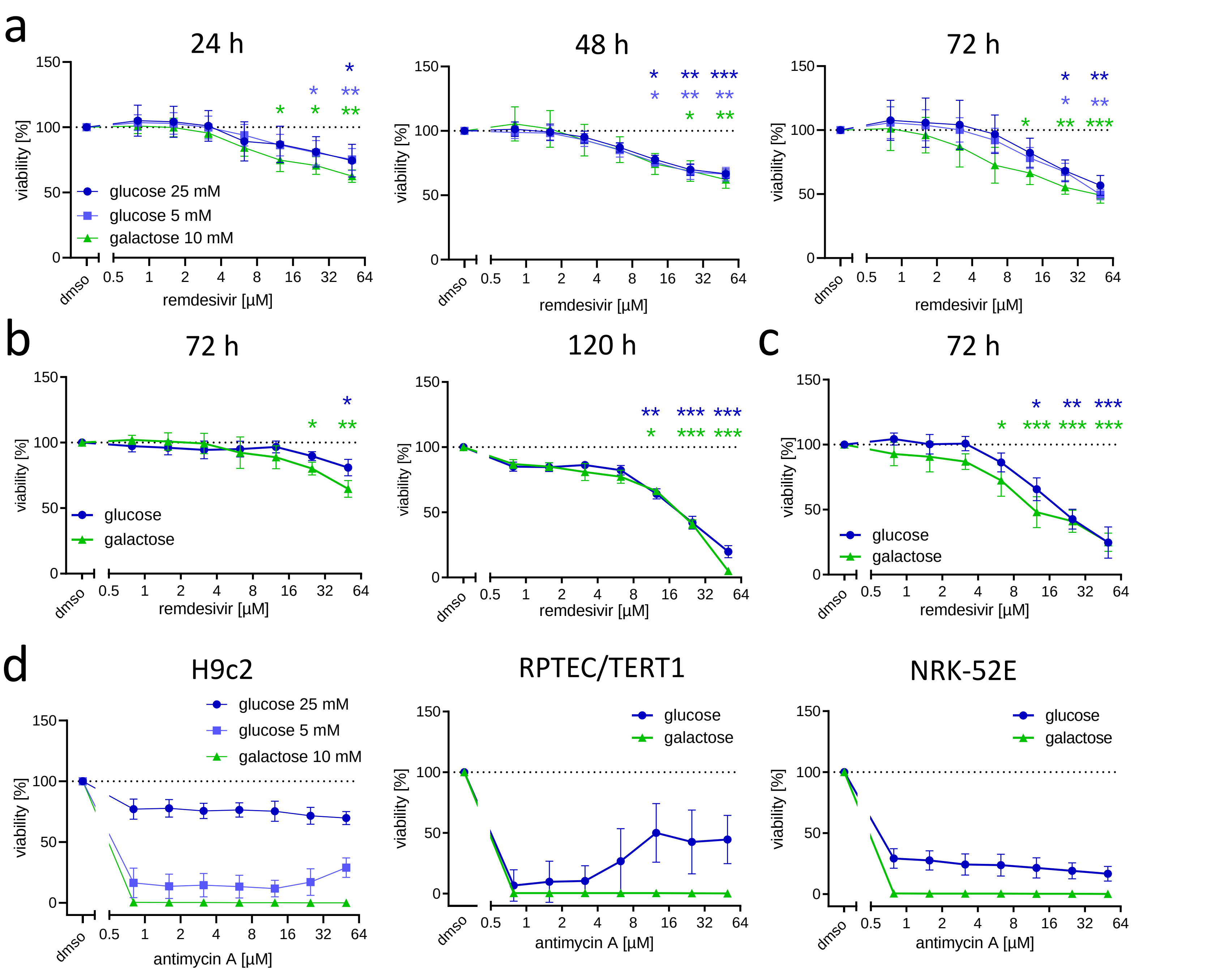

Fig. S6 The effect of different metabolic condtions on the toxicity of remdesivir and antimycin A Cells were treated with increasing concentrations of remdesivir (**a - c**) or antimycin A (**d**) and viability was determined by ATP-measurement after indicated time periods. (**a**) H9c2 cells were cultured and treated in medium containing 25 mM glucose, 5 mM glucose or 10 mM galactose (n = 5). (**b**) RPTEC/TERT1 cells were cultured and treated in medium containing 10 mM glucose or 10 mM galactose. Within the 120h period cells were retreated once (n = 5-7). (**c**) NRK-52E cells were cultured and treated in medium containing 25 mM glucose or 10 mM galactose (n = 7). (**d**) Indicated cell lines were treated with increasing concentrations of antimycin A for 72 h (H9c2: n = 3, RPTEC: n = 7, NRK-52E: n = 6) Data were normalized to the solvent controls (dmso = 100 %, dashed line). Kruskal-Wallis-test with Dunn‘s multiple comparisons test * $p < 0.05$, ** $p < 0.01$, *** $p < 0.001$

Figure S7

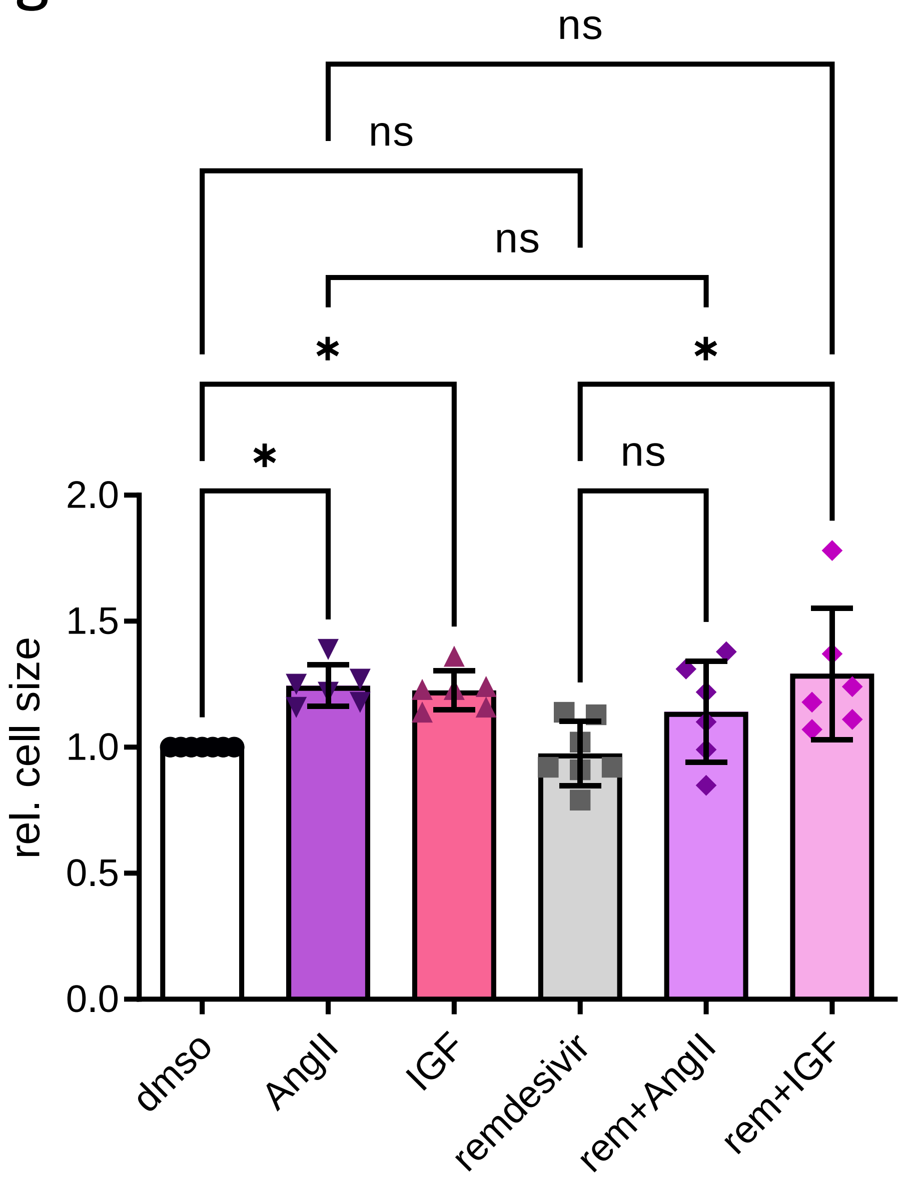

Fig. S7 Effect of remdesivir on the hypertrophic response of NMCM

NMCM were treated with 200 nM angiotensin II (AngII) or 300 pM insulin-like growth factor (IGF) with or without co-treatment with 6.25 µM remdesivir for 24 h. Cell sizes were determined by microscopical examination and normalized to the solvent control (dmso = 1). Kruskal-Wallis-test with Dunn‘s test for multiple comparisons ($n = 6$; * $p < 0.05$)

Figure S8

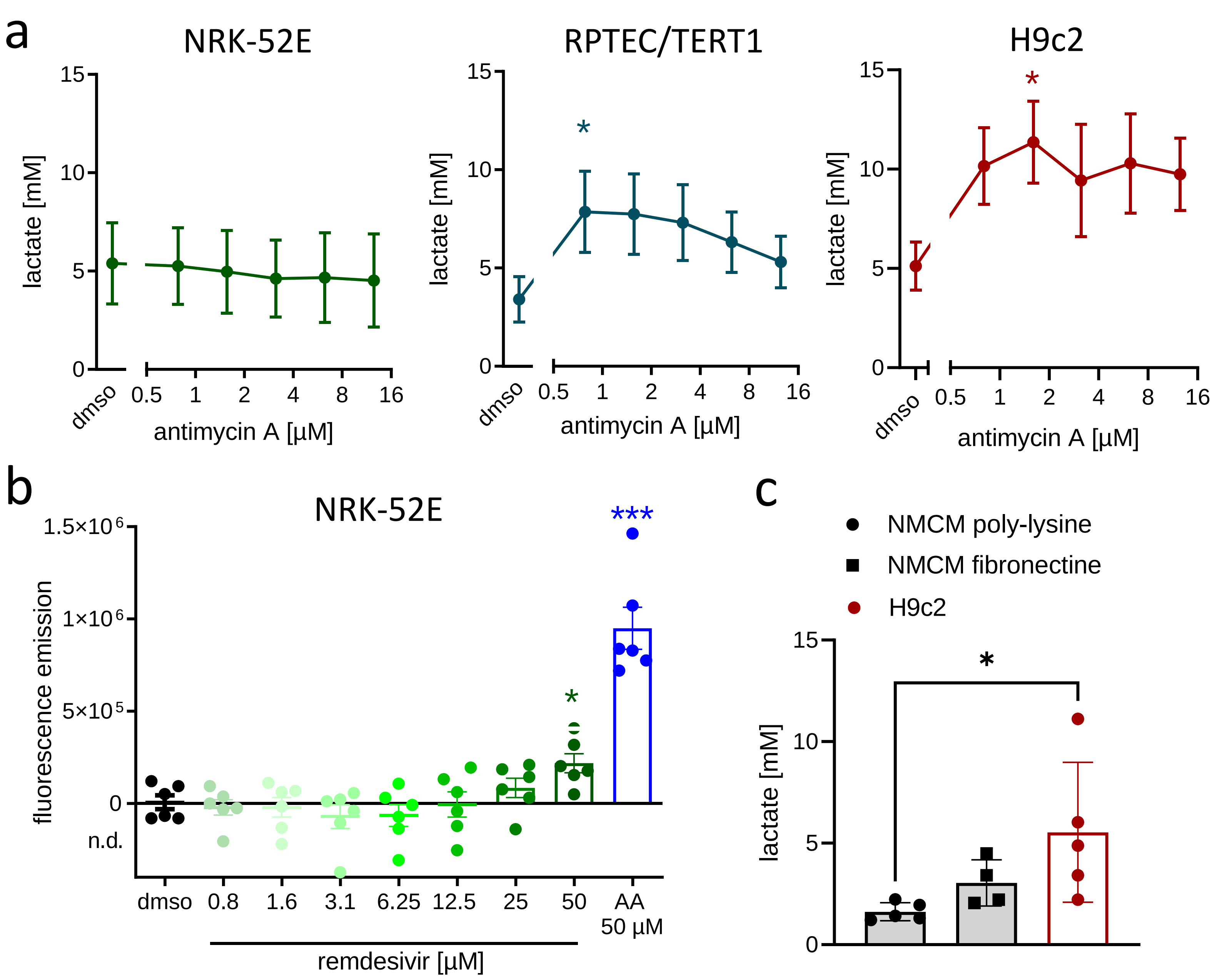

Fig. S8 Comparison of the relative sensitivity towards mitotoxic agents between different cell lines

(**a**) NRK-52E (n = 9), RPTEC/TERT1 (n = 7) or H9c2 cells (n = 3) were treated with increasing doses of antimycin A or solvent control (dmso) for 24 h and lactate was quantified in the supernatants. (**b**) NRK-52E cells were treated with remdesivir, antimycin A (AA) or solvent control (dmso) for 24 h and ROS-release was quantified in the supernatants by fluorescent detection of H_2O_2 (n = 6). (**a**, **b**) RM-ANOVA with Dunnet‘s multiple comparisons test * $p < 0.05$, *** $p < 0.001$ (**c**) indicated cell types were treated with 0.1 % dmso for 24 h. Lactate concentrations were determined in supernatants. *Data are also shown in a normalized way in Fig. 3 a (H9c2) and Fig. 3 c, e (NMCM)*. One-way ANOVA * $p < 0.05$
